# Rhenium Complexes Bearing Tridentate and Bidentate Phosphinoamine Ligands in the Production of Biofuel Alcohols via the Guerbet Reaction

**DOI:** 10.1021/acs.organomet.1c00313

**Published:** 2021-08-04

**Authors:** Ashley M. King, Richard L. Wingad, Natalie E. Pridmore, Paul G. Pringle, Duncan F. Wass

**Affiliations:** † Cardiff Catalysis Institute, School of Chemistry, Cardiff University, Main Building, Park Place, Cardiff CF10 3AT, United Kingdom; ‡ School of Chemistry, University of Bristol, Cantock’s Close, Bristol BS8 1TS, United Kingdom

## Abstract

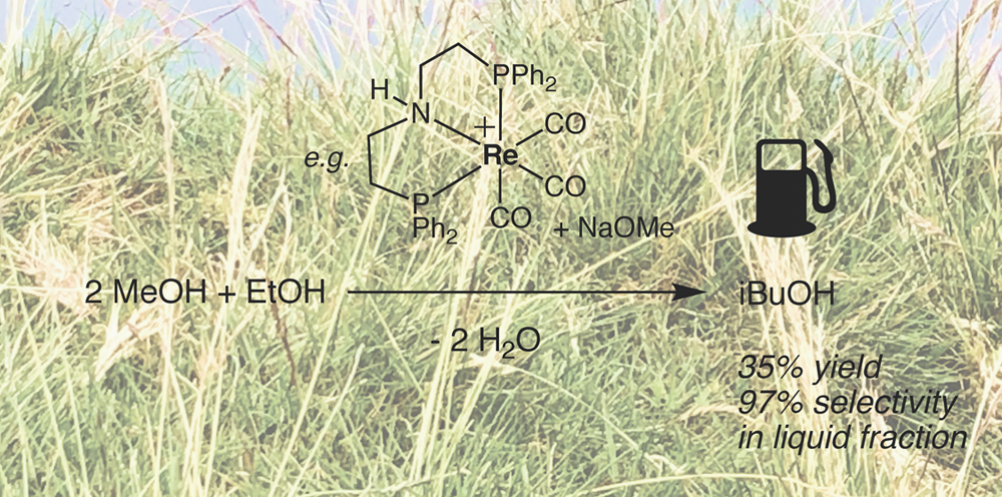

We report a variety of rhenium complexes supported by bidentate and tridentate phosphinoamine ligands and their use in the formation of the advanced biofuel isobutanol from methanol and ethanol. Rhenium pincer complexes **1**–**3** are effective catalysts for this process, with **2** giving isobutanol in 35% yields, with 97% selectivity in the liquid fraction, over 16 h with catalyst loadings as low as 0.07 mol %. However, these catalysts show poorer overall selectivity, with the formation of a significant amount of carboxylate salt solid byproduct also being observed. Production of the active catalyst **1d** has been followed by ^31^P NMR spectroscopy, and the importance of the presence of base and elevated temperatures to catalyst activation has been established. Complexes supported by diphosphine ligands are inactive for Guerbet chemistry; however, complexes supported by bidentate phosphinoamine ligands show greater selectivity for isobutanol formation over carboxylate salts. The novel complex **7** was able to produce isobutanol in 28% yield over 17 h. The importance of the N–H moiety to the catalytic performance has also been established, giving further weight to the hypothesis that these catalysts operate via a cooperative mechanism.

## Introduction

The production of sustainable liquid fuels is a key scientific and technological goal,^1^ biofuels offering a much cleaner alternative to liquid fossil fuels for use in transportation, if the appropriate feedstocks can be found.^2,3^ The most widely used biofuel is ethanol, accounting for over 50% of all biofuel production within the past decade.^4^ However, there are significant drawbacks to ethanol as a fuel, such as a lower energy density relative to gasoline, increased corrosion to current engine technology, and its hygroscopic nature leading to issues with phase separation.^5,6^ This limits blend ratios with regular gasoline, the use of higher blends requiring major engine modifications.^5^ Isomers of butanol have emerged as attractive alternatives to ethanol, since their physical properties are more similar to those of gasoline.^7,8^ Current commercial methods of butanol production include ABE fermentation, which suffers from selectivity issues and low yields, and the oxo process, which requires petrochemical feedstocks.^9−12^

**Scheme 1:**
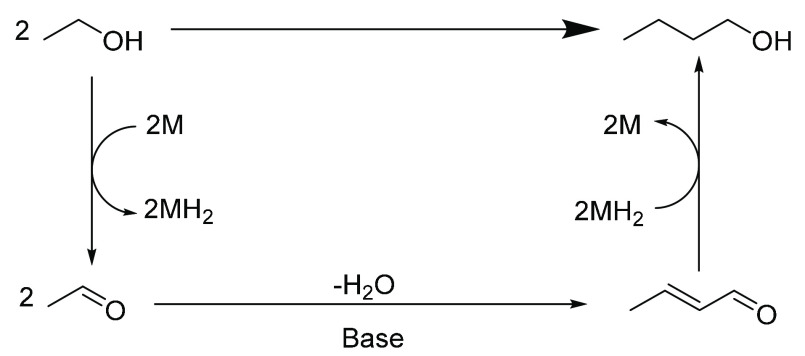
Guerbet Reaction^13^

**Figure 1:**
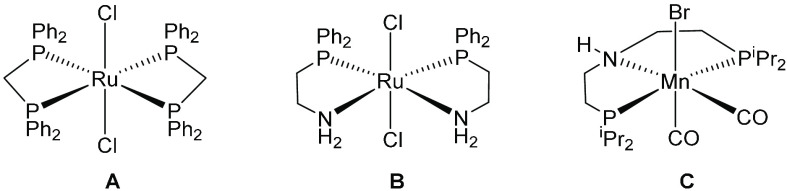
Some ruthenium- and manganese-based catalysts previously used for the formation of isobutanol from methanol and ethanol.^23,25,26^

More recently, the Guerbet reaction (Scheme 1) has emerged as a promising method to produce *n*-butanol from an ethanol feedstock.^14−19^ This 100-year-old reaction has been rediscovered in recent years, particularly in so-called borrowed hydrogen chemistry. Unfortunately, ethanol is a particularly challenging substrate for this reaction, in large part due to issues in controlling the reactivity of the intermediate acetaldehyde that can lead to poor selectivity. We, and others, have developed new catalysts that show good selectivity for this reaction, with many being based on ruthenium complexes: for example, **A** (Figure 1) produces *n*-butanol in a 9.6% yield with 94.1% selectivity and **B** produces *n*-butanol in a 17.1% yield with 93.5% selectivity over 4 h.^16,18^ Systems developed by Milstein and Szymczak show greater *n*-butanol yields (36% and 38%, respectively), but to the detriment of selectivity (68% and 84%, respectively) with longer chain alcohols being the major side products.^20,21^ Iridium catalysts have also been reported, including an early example by Ishii^15^ and a promising system using bulky inorganic bases by Baker and Jones.^22^ Here, the bulky base is believed to favor the coupling of acetaldehyde over longer-chain aldehydes and is responsible for an impressive selectivity of over 99%. The Guerbet reaction has also been extended to the formation of isobutanol (an isomer with preferred fuel characteristics) via the coupling of ethanol with two methanol molecules. Complex **A** gives exceptionally high conversion and selectivity for this reaction (66.4% and 98.1%, respectively, over 2 h).^23,24^

**Figure 2:**
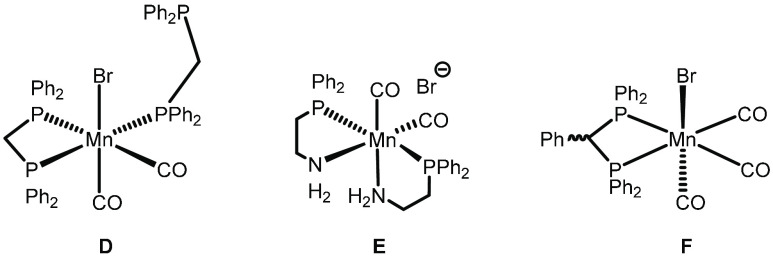
Manganese complexes used in the production of isobutanol.^28^

A recent focus has been on the development of Guerbet catalysts based on earth-abundant metals, manganese in particular receiving attention. Pincer complex **C** has been shown to be active for *n*-butanol formation in independent reports by the Jones and Liu groups.^25,26^ Liu reported *n*-butanol formation with very high turnover numbers (>100000) by extending run times to 168 h, with a very low catalyst loading (0.0001 mol %), and accepting low yields (9.8%). Under more practical conditions, Jones was able to generate 22% *n*-butanol over just 24 h. More recently, Liu has used the same catalyst for isobutanol formation, reporting yields of 40% in 96% selectivity over 48 h.^27^ We have reported the use of a variety of manganese complexes supported by bidentate ligands for isobutanol formation (Figure 2), with **F** giving yields of up to 21% over 90 h.^28^

**Figure 3:**
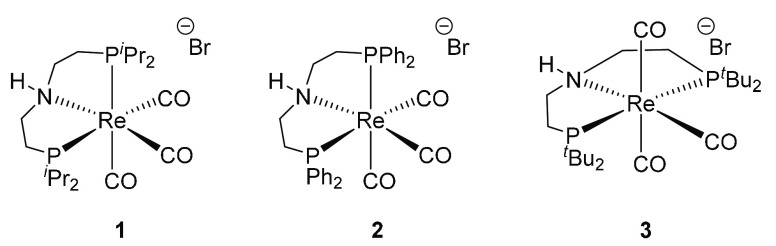
Rhenium catalysts used for hydrogenation and dehydrogenative coupling reactions and the *N*-methylation of anilines.^29−32^

Given the superior performance of ruthenium and iridium catalysts, and the growing number of studies of manganese systems, it seems surprising that rhenium catalysts for the Guerbet reaction have not been reported. There have been initial reports that complex **1** (Figure 3) will catalyze related dehydrogenative coupling and hydrogenation reactions.^29,30^ More recently, Sortais has reported that **1**–**3** are active for the *N*-methylation of anilines,^31^**2** producing the monomethylated product in 97% yield after 48 h.

We report here that rhenium complexes with tridentate and bidentate phosphinoamine ligands are effective catalysts for the production of biofuel alcohols via the Guerbet reaction.

## Results and Discussion

### Rhenium Pincer Complex Catalysis

Rhenium pincer complexes **1**–**3** (Figure 3) were synthesized using reported methods from [ReBr(CO)_5_] or [Re(CO)_3_(H_2_O)_3_]Br and the corresponding bis(phosphino)ethylamine ligand.^30,31^ Complex **1** was then used to establish optimal conditions for the production of isobutanol from ethanol and methanol (Table 1), using conditions adapted from earlier studies on ruthenium and manganese.

**Table 1:**
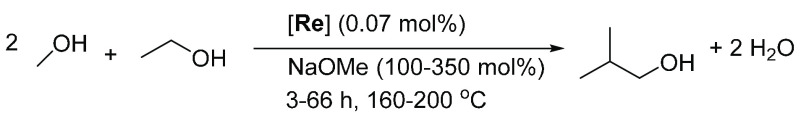
Production of Isobutanol by the Coupling of Ethanol and Methanol Using Rhenium Pincer Complexes

						liquid product			
entry^a^	catalyst	temp (°C)	time (h)	base loading (mol %)	EtOH conversn (%)	iBuOH yield (%)	nPrOH yield (%)	*i*BuOH slectivity (%)^b^	missing ethanol (%)^c^
1	**1**	180	18	200	96	16	2	85	75
2	**1**	180	66	200	99	21	0	99	77
3	**1**	180	3	200	73	5	3	40	56
4	**1**	180	18	100	85	10	4	56	65
5^d^	**1**	180	18	350					
6	**1**	160	18	200	79	7	4	48	61
7	**1**	200	18	200	100	15	0	97	84
8^e^	**1**	180	18	200	98	17	1	90	78
9	**2**	180	18	200	99	35	1	97	62
10	**3**	180	18	200	30	7	2	59	16

a Conditions: 1 mL (17.13 mmol) of EtOH, 10 mL of MeOH.

b Selectivity calculated from observed products in the liquid fraction (note the discrepancy between ethanol conversion and yield of liquid products because of solid products; see text).

c Missing ethanol is the discrepancy between ethanol conversion and the yield of liquid products.

d Large amount of solid produced.

e 0.1 mol % of the catalyst.

Complex **1** produced isobutanol in a 16% yield over 18 h (Table 1, entry 1) with 85% selectivity (defined as selectivity to the desired product in the liquid fraction), the main side product being the intermediate species *n*-propanol. It is clear from this first run that there is a large discrepancy in the mass balance of the reaction between ethanol conversion (96%) and total yield of liquid products; this is accounted for by the large amount of solid product that is obvious at the end of the run. This solid was isolated and analyzed by ^1^H and ^13^C NMR spectroscopy; both sodium formate and sodium acetate, in approximately a 14:1 molar ratio, were present. Given that the initial molar ratio of methanol to ethanol in the prereaction mixture is 14.4:1, it appears that the specific mixture of solid carboxylate formed is simply a function of the molar ratio of starting alcohols. Such solids are well-known as side products in Guerbet catalysis (even if they are not always reported) and form via the Cannizzaro or Tishchenko reaction from formaldehyde or acetaldehyde.^33,34^ Any formate produced can be further dehydrogenated to sodium carbonate, which is detected by ^13^C NMR spectroscopy. This dehydrogenation also produces hydrogen, which leads to a buildup of pressure within the autoclave over the course of the reaction.^18,24^ It seems that this rhenium catalyst has a particular propensity to form such solids in comparison to analogous manganese or ruthenium catalysts. Increasing the reaction time to 66 h serves to increase the yield of isobutanol as the intermediate *n*-propanol is converted (entry 2). However, shortening the reaction time to 3 h led to a significant reduction in yield with only 5% of isobutanol being observed (entry 3), albeit still with high levels of solid products (73% ethanol conversion). Decreasing the base loading and temperature was detrimental to both the yield and selectivity (entries 4 and 6). For the analogous manganese complex, the use of 350 mol % of NaOMe was reported to give the greatest isobutanol yields.^27^ However, with complex **1**, using elevated base loadings was found simply to increase the amount of solid produced, to the point where analysis of the postreaction mixture became impossible (entry 5). Increasing the reaction temperature to 200 °C led to complete ethanol conversion and an increase in isobutanol selectivity from 85% to 97% but was slightly detrimental to overall isobutanol yield (entry 7). Using an elevated catalyst loading of 0.1 mol % gave a slightly more favorable performance (entry 8) with 90% isobutanol selectivity and 98% ethanol conversion.

Complex **2**, containing PPh_2_ groups, displayed significantly improved performance in comparison to **1**, producing 35% isobutanol over an 18 h run time with excellent (97%) selectivity in the liquid fraction (Table 1, entry 9), although ethanol conversion was still significantly higher (99%). It is noteworthy that for manganese pincer catalysts, in contrast to this result, PPh_2_ donors give poorer performance relative to the P^*i*^Pr_2_ analogues.^27^ The performance of the P^*t*^Bu_2_ analogue **3** is inferior (entry 10); the origin of the superior performance of complex **2** is currently unclear, although it appears that the greater the steric bulk and electron-donating properties of the phosphine substituents, the less effective the complex is for the rhenium-catalyzed Guerbet reaction.

**Scheme 2:**
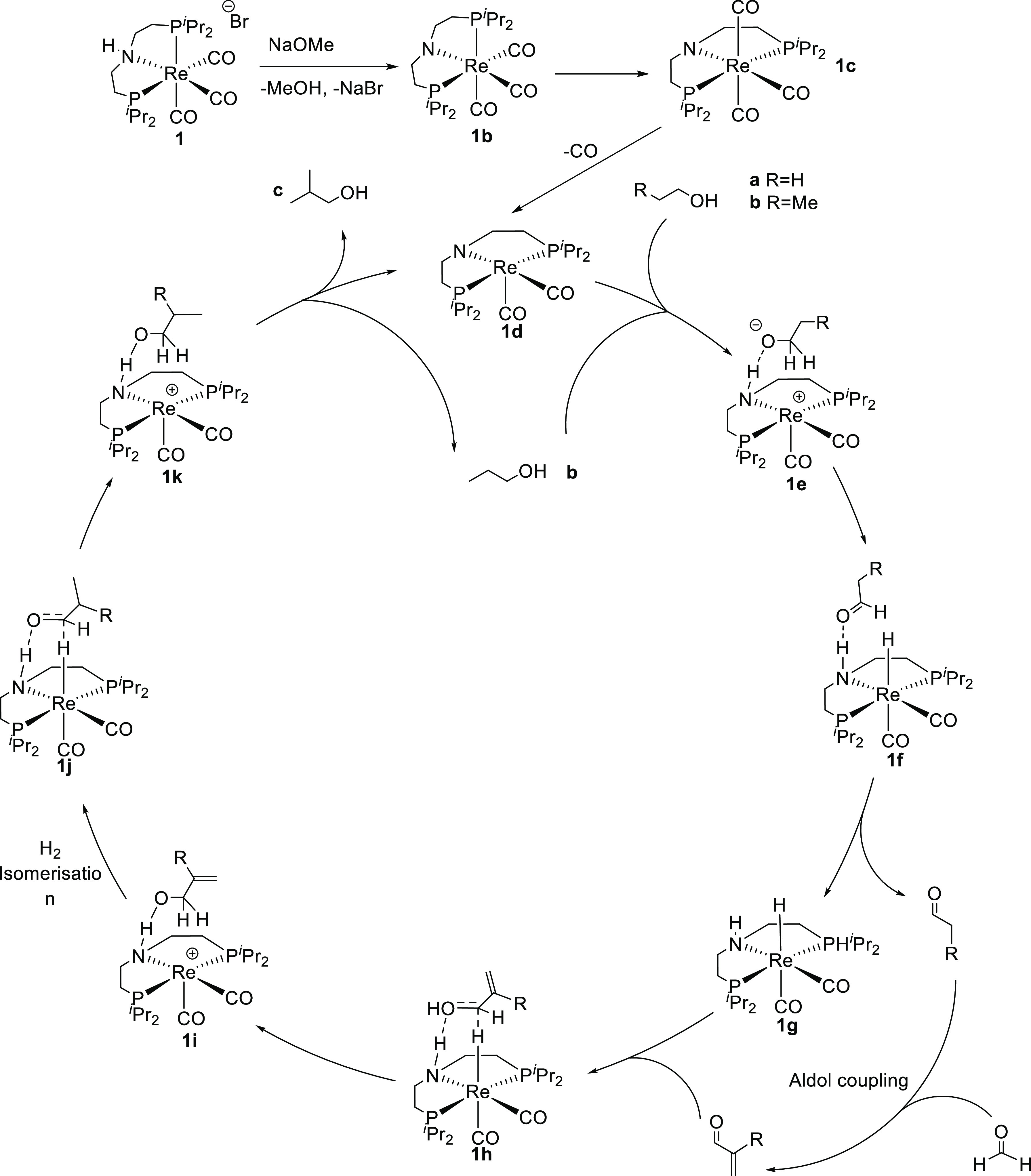
Proposed Catalytic Cycle for the Production of Isobutanol, Including the Formation of the Active Catalyst **1d**, on the Basis of Computational Work Performed by Sortais et al.^31^

Pincer complexes such as **1** have now been established to operate via an outer-sphere or cooperative mechanism, where the ligand amine moiety is an integral part of the catalyst active site.^30,31^ An analogous mechanism, supported by computational studies, is proposed for the *N*-methylation of anilines catalyzed by rhenium pincer complexes.^31^ This has been adapted to provide a possible mechanism for the production of isobutanol using complex **1** (Scheme 2). The precatalyst **1** must undergo transformation before the active catalyst (**1d**) is formed; while the formation of this species has been studied computationally, experimental spectroscopic evidence for the formation of species **1b** or **1c** has not been reported. The formation of **1b** and **1c** is predicted to be energetically favored, and carbonyl dissociation to form **1d** is only slightly energetically uphill, indicating that the formation of the active catalyst should be rapid and facile. Once the active catalyst **1d** is formed, it reacts with ethanol (**a**) via species **1e**, which contains a hydrogen bond between the EtOH and the deprotonated amine in the ligand backbone. Dehydrogenation then occurs, forming acetaldehyde and the rhenium hydride complex **1g**. Acetaldehyde reacts with formaldehyde, produced via the same mechanism with methanol as the substrate, in an aldol condensation to give acrylaldehyde. This α,β-unsaturated species reacts with **1g** via the C=O bond (as shown in Scheme 2, **1h** and **1i**); this then undergoes isomerization, and hydrogen is re-added to the rhenium complex (**1j**). Finally, the re-formed C=O bond is hydrogenated again (**1k**) and *n*-propanol (**b**) is produced. *n*-Propanol can subsequently re-enter the cycle, coupling with a further molecule of methanol (via formaldehyde) to generate isobutanol (**c**), which does not undergo further aldol condensation. Since formaldehyde cannot undergo an aldol condensation with itself, maintaining a high concentration of methanol vs ethanol suppresses *n*-butanol formation via ethanol self-condensation. Both substrate dehydrogenation and (re)hydrogenation are proposed to occur in the outer sphere of the catalyst.

The addition of 100 equiv of sodium methoxide to a methanol solution of **1** gave little change in the ^31^P{^1^H} NMR spectrum, even after heating to reflux for 20 h (see Figure S15 in the Supporting Information). Heating a methanol solution of **1** to 180 °C in an autoclave in the absence of base yielded a complex mixture of unidentified products (Figure S18). However, there was no indication that the active catalyst had been formed. Sodium formate, from the dehydrogenation of methanol, was observed in the postreaction mixture when a methanol solution of **1** was heated to 180 °C for 18 h with a 1000-fold excess of sodium methoxide, confirming that the active catalyst had been produced. The postreaction mixture also showed a singlet at 53.9 ppm in the ^31^P{^1^H} NMR spectrum; this has been tentatively assigned to complex **1d** (Figure S16). It appears that both base and elevated temperatures are required in order to produce the active catalyst from **1**.

### Rhenium Complexes Supported by Bidentate Ligands

**Figure 4:**
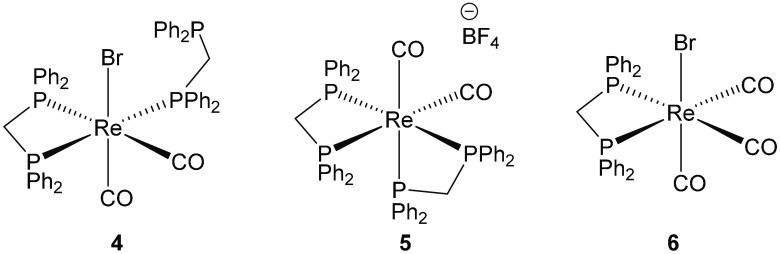
Rhenium complexes containing diphosphine ligands.

We have detailed the use of manganese bis chelate and bidentate catalysts for isobutanol formation, demonstrating that tridentate complexes are not a prerequisite for activity.^28^ Given these results, it appeared that analogous rhenium complexes would be compelling targets as potential catalysts. Complexes **4**–**6** (Figure 4) containing bis(diphenylphosphino)methane (dppm) ligands were prepared according to literature procedures.^35−37^

**Scheme 3:**
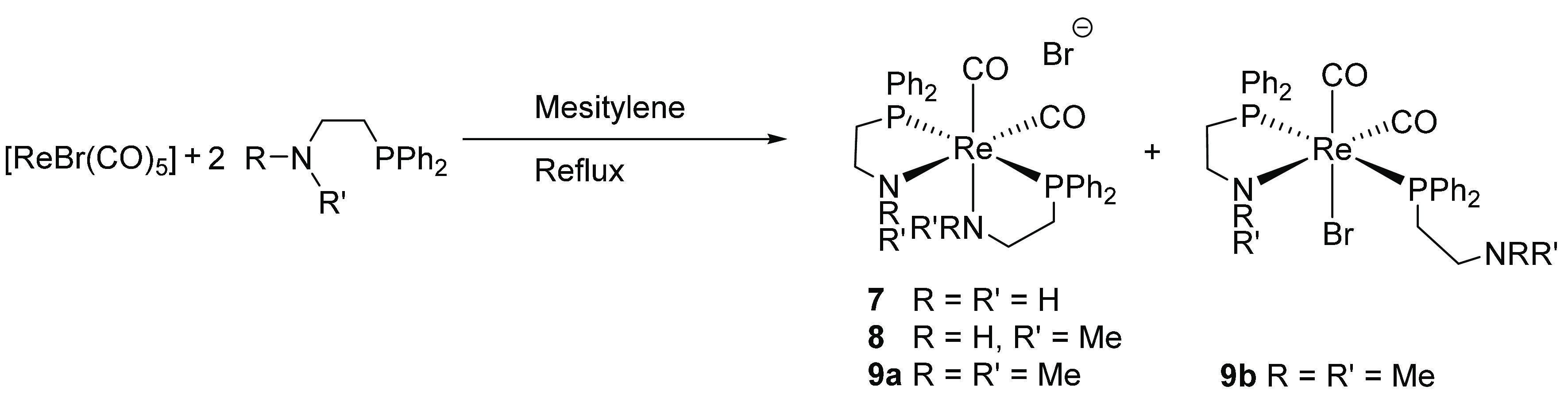
Formation of Rhenium Bis Chelates Bearing Phosphinoamine Ligands

The novel rhenium complexes **7**–**9** bearing phosphinoamine ligands were synthesized from a 2:1 solution of the ligand and [ReBr(CO)_5_] in refluxing mesitylene in 62%, 31%, and 5% yields, respectively (Scheme 3). Complexes **7** and **8** show singlets in their ^31^P{^1^H} NMR spectra at 41.3 and 38.0 ppm, respectively. Formation of the dicarbonyl species was confirmed by mass spectrometry, and a *cis* geometry was established by the observation of two peaks in the carbonyl region of the IR spectrum (see the Supporting Information for further details). When 2-(diphenylphosphino)-*N*,*N*-dimethylethylamine was used as the ligand under the same reaction conditions, a more complex mixture of the dicarbonyl complex **9a** and the pendant-ligand complex **9b** was formed. The ^31^P{^1^H} NMR spectrum of complex **9a** showed a singlet at 28.8 ppm, and for **9b** two doublets were observed at 32.2 and 4.2 ppm with a ^2^*J*_PP_ coupling of 210 Hz, indicating a *trans* phosphine coordination. Single crystals of **7** were obtained by layering a methanol solution with diethyl ether. The X-ray crystal structure of **7** shows a slightly distorted octahedral geometry with *trans* phosphine ligands and *cis* carbonyl ligands, in line with the analogous manganese complex (Figure 6).^38^

**Figure 5:**
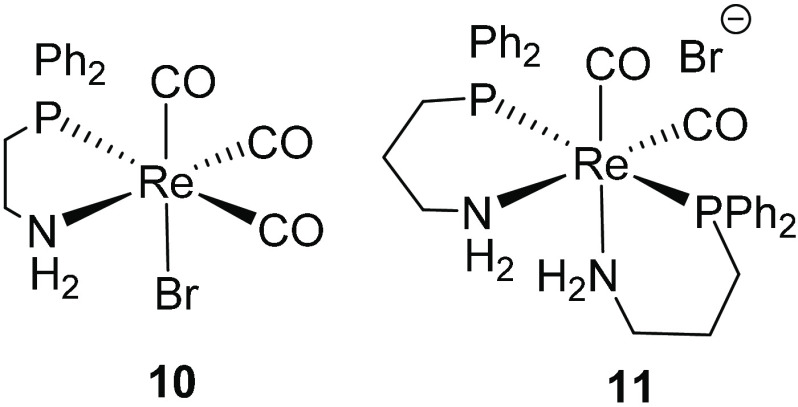
Rhenium complexes bearing dppea or dpppa ligands.

**Figure 6:**
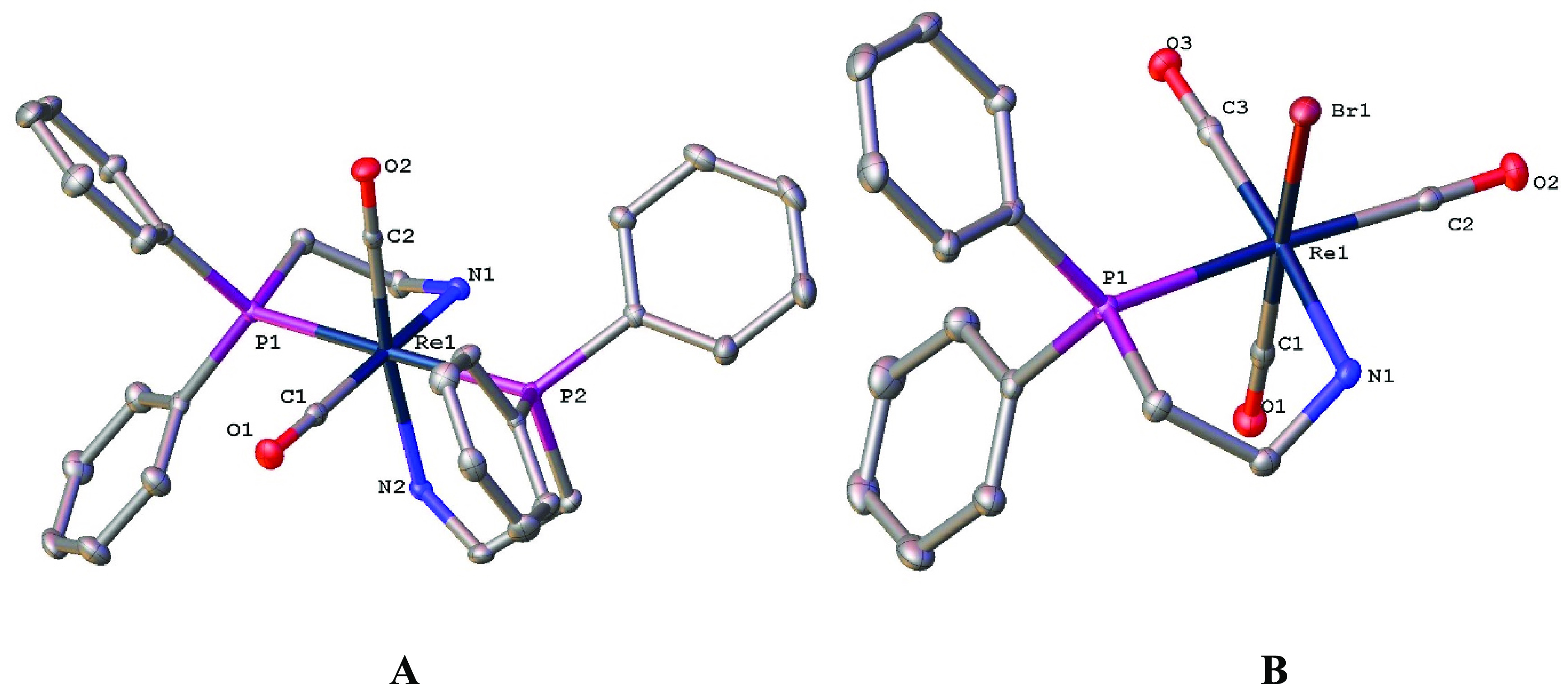
X-ray crystal structures of complexes **7** (A) and **10** (B). Ellipsoids are depicted at the 50% probability level. Hydrogen atoms, solvent molecules, and, in the case of **7**, the bromide counterion have been omitted for clarity.

The mono chelate dppea complex **10** and the bis chelate dpppa complex **11** (Figure 5) were prepared in 57% and 77% yields, respectively, from a 1:1 or 2:1 mixture of the ligand with [ReBr(CO)_5_]. Single crystals of complex **10** were grown by layering a dichloromethane solution with hexane. The X-ray crystal structure of **10** revealed an octahedral geometry with the bromide ligand in a *cis* orientation to the dppea ligand (Figure 6); again this is analogous to the structure of the equivalent manganese complex.^38^ Single crystals of **11** were grown by layering a dichloromethane solution with diethyl ether. The X-ray crystal structure again revealed a distorted-octahedral geometry with *trans* phosphine ligands and *cis* carbonyl ligands, analogous to **7** (see the Supporting Information for further details).

Complexes **4**–**11** were tested for activity in ethanol/methanol conversion to isobutanol using the conditions established previously; the results are shown in Table 2. Complexes **4**–**6** show little activity for isobutanol production over 17 h (entries 1–3). We have reported in stoichiometric studies that the catalytically active analogous manganese complex undergoes ligand redistribution reactions to give a mixture of the *cis* and *trans* bis chelate complexes, free ligand, and a mono chelate species.^28^ In contrast, treatment of **4** with 100 equiv of NaOMe in refluxing methanol cleanly produces a *cis* bis chelate complex analogous to **5** that is then stable under these conditions for at least 3 days. Similarly, complex **5** is stable under the same conditions. This increased stability is as expected in moving from the first-row metal to its third-row congener and suggests precatalytic reactions for manganese, which are not observed for rhenium and could be important in the observed differences in performance between these complexes. Complex **6** is also inactive for isobutanol production.

**Table 2:**
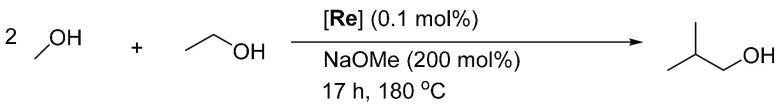
Production of Isobutanol by the Coupling of Ethanol and Methanol Using Rhenium Complexes Bearing Bidentate Ligands

			liquid product					
entry^a^	catalyst	EtOH conversn (%)	*i*BuOH yield (%)	*n*PrOH yield (%)	iBuOH selectivity (%)^b^	TON	TOF (h^–1^)	missing ethanol (%)^c^
1	**4**		trace					
2	**5**		trace					
3	**6**		trace					
4	**7**	52	12	7	58	124	7.3	28
5^d^	**7**	81	28	4	88	283	16.7	49
6	**8**	40	9	4	70	88	5.2	27
7	**9**	1						
8	**10**	26	6	5	45	59	3.5	10
9	**11**		trace					

a Conditions unless specified otherwise: 1 mL (17.13 mmol) of EtOH, 10 mL of MeOH, 180 °C, 17 h, NaOMe (200 mol %), 0.1 mol % of [Cat.].

b Selectivity calculated from observed products in the liquid fraction.

c Missing ethanol is the discrepancy between ethanol conversion and the yield of liquid products.

d 200 °C.

Complex **7**, bearing mixed donor phosphinoamine ligands, is active for isobutanol formation, producing 12% isobutanol with 52% ethanol conversion over 17 h at 180 °C (Table 2, entry 4). These results are comparable with those for the pincer complex **1** (Table 1, entry 1), albeit the selectivity to isobutanol in the liquid fraction is lower (58%) due to a significant amount of the intermediate *n*-propanol; 2-methylbutanol from the coupling of propanol and ethanol is also observed in greater amounts in this case (5% yield in the liquid fraction). A significant advantage of this catalyst over complexes **1**–**3** is that the mass balance to liquid products is improved; the amount of solid carboxylate products is reduced. Raising the reaction temperature to 200 °C (Table 2, entry 5), increased both the isobutanol yield and selectivity to 28% and 88%, respectively, although the ethanol conversion also increased (81%). It is instructive to compare this to the analogous manganese complex ,which produced 9% isobutanol over 90 h under the same conditions; this corresponds to a 17-fold increase in TOF.^28^ At 180 °C complex **8** shows similar performance to **7** (Table 2, entry 6). Conversely, **9** is completely inactive for isobutanol production, demonstrating the importance of the N–H moiety for catalytic activity (Table 2, entry 7). This supports the hypothesis that rhenium phosphinoamine catalysts may operate via a cooperative mechanism, as has been postulated for their ruthenium analogues.^18^ Examples of rhenium complexes using a ligand-assisted mechanism for the *N*-monomethylation of anilines have been reported.^31^ Complex **10** has poorer performance (Table 2, entry 8) than complex **9**, and complex **11** is inactive within error (Table 2, entry 9).

## Conclusion

A variety of rhenium complexes have been synthesized and used for the catalytic upgrading of ethanol and methanol to isobutanol, demonstrating that complexes of this metal are competent catalysts for the Guerbet reaction. Rhenium pincer complexes are active for this conversion, with complex **2** giving the best performance of 35% isobutanol over 18 h. An analysis of the full mass balance of reaction products reveals that these complexes also produce large amounts of sodium formate and sodium acetate solid byproducts, meaning the overall selectivity for ethanol conversion to isobutanol is low. A variety of rhenium complexes of bidentate ligands were also tested. Of these, complex **8** gave an isobutanol yield of 28% and significantly less solid byproduct. Comparisons to other Mn, Ru, or Ir catalysts are revealing but challenging, since the key issue of overall selectivity including solid products is often not reported. However, two broad trends can be observed: first, rhenium catalysts outperform isostructural manganese systems in terms of isobutanol yield, but second, this is at the expense of a higher propensity to produce sodium carboxylate side products in comparison to other catalysts. The reasons for this are likely to be the subtle interplay of rates for competing Guerbet, Cannizzaro, and Tishchenko reactions across the various systems.

## Supporting Information Available

Full experimental procedures, characterization data, detailed catalytic results, and crystallographic data (PDF)

## Accession Codes

CCDC 2072199–2072201 contain the supplementary crystallographic data for this paper. These data can be obtained free of charge via www.ccdc.cam.ac.uk/data_request/cif, or by emailing data_request@ccdc.cam.ac.uk, or by contacting The Cambridge Crystallographic Data Centre, 12 Union Road, Cambridge CB2 1EZ, UK; fax: +44 1223 336033.
