# Supplementary material for: Rhenium Complexes Bearing Tridentate and Bidentate Phosphinoamine Ligands in the Production of Biofuel Alcohols via the Guerbet Reaction
Source: Organometallics. 2021 Aug 4;40(16):2844–51. doi: 10.1021/acs.organomet.1c00313 (PMC8411595; doi:10.1021/acs.organomet.1c00313)
Supplement: Supplementary file 1 — om1c00313_si_001.pdf [file om1c00313_si_001.pdf]

Rhenium complexes bearing tridentate and bidentate phosphinoamine ligands in the production of biofuel alcohols via the Guerbet reaction

Ashley M. King[a], Richard L. Wingad[a], Natalie E. Pridmore[b], Paul G. Pringle[b] and Duncan F. Wass[a]*

[a] Cardiff Catalysis Institute, School of Chemistry, Cardiff University, Main Building, Park Place, Cardiff, CF10 3AT, United Kingdom

[b] School of Chemistry, University of Bristol, Cantock's Close, Bristol, BS8 1TS, United Kingdom

Supporting Information

Contents

Experimental details……S2

General considerations……S2

Complex Synthesis……S2

Catalysis……S5

Mechanistic experiments……S7

Spectra……S9

Crystallographic data……S19

References……S21

Experimental details

General considerations

All procedures were carried out under an inert atmosphere (N_2) using standard Schlenk line techniques or in an inert atmosphere glovebox (Ar). Bis(diphenylphosphino)methane, 3-(diphenylphosphino)-1-propylamine, [ReBr(CO)$_5$], bis[(2-diisopropylphosphino)ethyl]amine solution (10 wt% in THF), bis[2-(di-*tert*-butylphosphino)ethyl]amine solution (10 wt% in THF) and bis[(2-diphenylphosphino)ethyl]ammonium chloride were purchased from Sigma Aldrich and used without further purification. Solvents were purified using an anhydrous Engineering Grubbs-type solvent system, except for anhydrous ethanol and methanol which were purchased from Sigma-Aldrich and used as received. 2-(diphenylphosphino)ethylamine,[1] **1**,[2] **3**,[3] [Re(H$_2$O)$_3$(CO)$_3$]Br,[4,5] **4**,[6] **5**[7] and **6**[8] were synthesised *via* literature procedures. 2-(diphenylphosphino)-N-methylethylamine was kindly supplied by Dr H. Aitchison and was prepared by a modified literature proccdure.[1] 2-(diphenylphosphino)-N,N-dimethylethylamine was prepared *via* a modified literature procedure.[1] NMR spectra were recorded on Jeol ECS300, Varian 400MR and Bruker 500 NMR spectrometers. ^{1}H NMR chemical shifts were referenced relative to the residual solvent resonances in the deuterated solvent or carried out unlocked. ^{31}P{^{1}H} NMR spectra were referenced relative to 85% H_3PO_4 external standard. Infrared spectra were recorded on a Perkin Elmer Spectrum Two FT-IR spectrometer as solid samples in air. Mass spectra (Nanospray) were recorded on a Thermo Scientific Orbitrap Elite.

Complex synthesis

Synthesis of 2- [(Re(CO)$_3$(PNP^{Ph})]Br

Bis[(2-diphenylphosphino)ethyl]ammonium chloride (0.1 g, 0.209 mmol) was suspended in toluene (4 mL) in a round bottom flask. NaOH (2 mL, 10 wt% in water) was added sequentially while stirring until all starting material has dissolved. The aqueous layer was then removed, and the organic layer was washed with distilled water (3 x 2 mL) and dried over sodium sulphate. Toluene was removed *in vacuo* giving bis[(2-diphenylphosphino)ethyl]amine as a clear oil.

[Re(CO)$_3$(H$_2$O)$_3$]Br (0.081 g, 0.200 mmol) was measured out in a Schlenk flask and suspended in dichloromethane (2 mL). The ligand was dissolved in DCM (4 mL) and added dropwise to the complex while stirring, the Schlenk was then washed through with DCM (2 mL). The suspension was heated to 40 ^{o}C for 18 hours, giving a colourless liquid and a fine grey

suspension. The solution was filtered, reduced in volume by approx. 50%, and triturated with pentane giving a fine white powder. This was isolated by filtration and dried *in vacuo* (0.072 g, 45.5%). The NMR data was consistent with the literature.[3]

$^{31}P\{^{1}H\}$ NMR (122 MHz, $CDCl_3$): (δ, ppm) 23.09

Synthesis of 7- [Re(CO)$_2$(dppea)$_2$]Br

A solution of 2-(diphenylphosphino)ethylamine (0.225 g, 0.985 mmol) in mesitylene (8 mL) was added slowly to a stirred suspension of [ReBr(CO)$_5$] (0.2 g, 0.492 mmol) in mesitylene (8 mL). This was stirred at room temperature for 5 minutes before heating to reflux for 18 hours. Upon heating the suspension dissolved to give a clear, colourless solution. After reflux, a pale-yellow solution and an off-white solid were observed, the solution was cooled in an ice-water bath causing further precipitation. The solid was isolated by filtration, washed with hexane (3 x 8 mL), and dried *in vacuo* giving an off-white powder (0.305 g, 62%). Single crystals for use in X-ray diffraction were grown from a layered solution of methanol with Et_2O.

$^{31}P\{^{1}H\}$ NMR (122 MHz, $CDCl_3$): (δ, ppm) 41.28

^{1}H NMR (300 MHz, $CDCl_3$): (δ, ppm) 7.83 (br, m, 4H), 7.64 (br, m, 4H), 7.50 (br, m, 12H), 5.69 (br, t, 2H), 3.73 (br, q, 2H), 2.90 (br, t, 2H), 2.69 (br, s, 2H), 2.43 (br, s, 2H), 2.19 (br, s, 2H)

HR MS (Nanospray): *m/z* calc for $[M]^+$ $C_{30}H_{32}ReN_2O_2P_2$ 701.1497 Found 701.1491 (0.9 ppm)

IR (ν, cm^{-1}) 1920, 1834

Synthesis of 8- [Re(CO)$_2$(Me-dppea)$_2$]Br

A solution of 2-(diphenylphosphino)-N-methylethylamine (0.120 g, 0.492 mmol) in mesitylene (4 mL) was added slowly to a stirred suspension of [ReBr(CO)$_5$] (0.100 g, 0.246 mmol) in mesitylene (4 mL). This was stirred at room temperature for 10 minutes before heating to reflux for 60 hours. Upon heating the suspension dissolved to give a clear, colourless solution. After reflux, an off-white suspension was observed. The solid was isolated by filtration, washed with hexane (3 x 8 mL), and dried *in vacuo* giving an off-white powder (0.062 g, 31%).

$^{31}P\{^{1}H\}$ NMR (122 MHz, $CDCl_3$): (δ, ppm) 38.03

^{1}H NMR (300 MHz, $CDCl_3$): (δ, ppm) 7.88 (br, m, 4H), 7.65 (br, m, 4H), 7.50 (br, m, 6H), 7.36 (br, m, 6H), 5.72 (br, s, 2H), 3.36 (br, m, 4H), 2.72 (br, m, 2H), 2.56 (br, m, 2H), 1.93 (s, 3H), 1.85 (s, 3H)

HR MS (Nanospray): *m/z* calc for $[M]^+$ $C_{32}H_{36}ReN_2O_2P_2$ 729.1810 Found 729.1805 (0.7 ppm)

IR (*ν*, cm^{-1}) 1921, 1835

Synthesis of 9a/9b- [Re(CO)$_2$(Me$_2$-dppea)$_2$]Br

A solution of 2-(diphenylphosphino)-N,N-dimethylethylamine (0.127 g, 0.492 mmol) in mesitylene (4 mL) was added slowly to a stirred suspension of $[ReBr(CO)_5]$ (0.1 g, 0.246 mmol) in mesitylene (4 mL). This was stirred at room temperature for 10 minutes before heating to reflux for 90 hours. Upon heating the suspension dissolved to give a clear, colourless solution. The solution was allowed to cool to room temperature, filtered and triturated with hexane (30 mL). The precipitate was isolated *via* filtration and dried *in vacuo* (0.022 g, 5.4%).

$^{31}P\{^{1}H\}$ NMR (122 MHz, $CDCl_3$): (δ, ppm) 32.24 (d, $^{2}J_{PP}$= 209 Hz), 28.76 (s), 4.17 (d, $^{2}J_{PP}$= 209 Hz)

^{1}H NMR (300 MHz, $CDCl_3$): (δ, ppm) 7.69 (br, m, 10H), 7.43 (br, m, 15H), 3.18 (s, 3H), 3.04 (s, 3H), 2.38 (s, 2H), 2.19 (br, s, 4H), 2.12 (s, 2H) (other methylene protons not visible)

HR MS (Nanospray): *m/z* calc for $[M]^+$ $C_{34}H_{41}ReN_2O_2P_2Br$ 837.1384 Found 837.1360 (-2.9 ppm) (HR MS calculated for complex **9b**)

IR (*ν*, cm^{-1}) 2020, 1916, 1882, 1827

Synthesis of 10- [ReBr(CO)$_3$(dppea)]

A solution of 2-(diphenylphosphino)ethylamine (0.113 g, 0.492 mmol) in toluene (7 mL) was added slowly to a stirred suspension of $[ReBr(CO)_5]$ (0.200 g, 0.492 mmol) in toluene (7 mL). The suspension was heated to reflux for 18 hours. Upon heating the suspension dissolved to give a clear, colourless solution. The solution was allowed to cool and toluene was removed *in vacuo*. The crude solid was dissolved in THF (12 mL) and filtered, giving a golden solution. The solution was reduced to 1.5 mL and triturated with hexane (20 mL). The off-white solid produced was isolated by filtration and dried *in vacuo* (0.147 g, 56.7%). Single crystals for use in X-ray diffraction were grown from layering a DCM solution with hexane.

$^{31}P\{^1H\}$ NMR (122 MHz, $CDCl_3$): (δ, ppm) 28.42

^1H NMR (300 MHz, $CDCl_3$): (δ, ppm) 7.72 (br, m, 2H), 7.58 (br, m, 2H), 7.42 (br, m, 6H), 3.91 (br, s, 1H), 3.17 (br, s, 1H), 2.75 (br, m, 2H), 2.35 (br, s, 1H)

HR MS (Nanospray): *m/z* calc for $[M-Br]^+$ $C_{17}H_{16}NO_3PRe$ 500.0425 Found 500.0410 (-3.0 ppm)

IR (ν, cm^{-1}) 2018, 1907, 1876

Synthesis of 11- $[Re(CO)_2(dpppa)_2]Br$

A solution of 3-(diphenylphosphino)propylamine (0.180 g, 0.739 mmol) in mesitylene (6 mL) was added slowly to a stirred suspension of $[ReBr(CO)_5]$ (0.150 g, 0.369 mmol) in mesitylene (6 mL), the ligand was washed through with further mesitylene (1 mL). This was stirred at room temperature for 5 minutes before heating to reflux for 18 hours. Upon heating the suspension dissolved to give a clear, colourless solution. After reflux, a colourless solution and a white solid were observed, the solution was cooled in an ice-water bath causing further precipitation. The solid was isolated by filtration, washed with hexane (3 x 7 mL), and dried *in vacuo* giving a white powder (0.206 g, 76.6%).

$^{31}P\{^1H\}$ NMR (122 MHz, $CDCl_3$): (δ, ppm) 12.47 (br s)

^1H NMR (300 MHz, $CDCl_3$): (δ, ppm) 7.79 (br m, 4H), 7.50 (br m, 16H), 5.22 (br t, 2H, $^2J_{PH}$ = 11.4 Hz), 3.14 (br m, 4H), 2.22 (br m, 8H), 1.90 (br m, 2H)

HR MS (Nanospray): *m/z* calc for $[M]^+$ $C_{32}H_{36}N_2O_2P_2Re$ 729.1810 Found 729.1818 (1.1 ppm)

IR (ν, cm^{-1}) 1913, 1806

Catalysis

Catalytic reactions were carried out in a 100 mL Parr stainless steel autoclave with aluminium heating mantle and using magnetic stirring. A typical procedure is shown below. Full catalytic results are shown in Tables S1 and S2

General catalytic procedure- isobutanol formation

A rhenium complex (0.012 mmol, 0.07 mol%) and sodium methoxide (1.85 g, 34.26 mmol, 200 mol%) were added to a clean oven-dried fitted PTFE insert equipped with a stirrer bar in

a glove box. The autoclave was sealed in a glove box and then put under a nitrogen atmosphere on a Schlenk line. Methanol (10 mL) was injected into the autoclave through an inlet against a flow of nitrogen followed by ethanol (1 mL, 17.13 mmol). The autoclave was sealed and placed into the pre-heated (180 °C) aluminium heating mantle. After the reaction run time, the autoclave was cooled to room temperature in an ice-water bath. The autoclave was vented to remove any gas generated during the reaction. A liquid sample was removed, filtered through a short plug of alumina (acidic) and analysed by GC (100 μL of sample, 10 μL of hexadecane standard, 1.7 mL Et_2O).

Table S1: Rhenium pincer complexes for the conversion of methanol and ethanol to isobutanol

**Run** [a]	**Complex (mol%)**[b]	**Base Loading (mol%)**	**Runtime (h)**	**Ethanol Conv. (%)**	**TON**[c] **(Selectivity)**[d] **[Yield]**		
					iBuOH	PrOH	2MeBuOH
**1**	**1**(0.07)	200	18	96	229(85)[16]	21(8)[1.5]	19(7)[3]
**2**	**1**(0.07)	200	66	99	300(99)[21]	0(0)[0]	4(1.5)[0.50]
**3**	**1**(0.07)	200	3	73	71(40)[5]	47(26)[3]	62(35)[9]
**4**	**1**(0.07)	100	18	85	142(56)[10]	60(25)[4]	45(19)[6]
**5**	**1**(0.07)	350	18	-	-	-	-
**6**[e]	**1**(0.07)	200	18	79	100(48)[7]	58(29)[4]	47(23)[7]
**7**[f]	**1**(0.07)	200	18	100	214(97)[15]	0(0)[0]	6(3)[1]
**8**	**1**(0.1)	200	18	98	170(90)[17]	7(4)[0.7]	10(6)[2]
**9**	**2**(0.07)	200	18	99	500(97)[35]	7(2)[0.6]	9(1)[1.2]
**10**	**3**(0.07)	200	18	30	100(59)[7]	29(19)[2]	36(23)[5]

[a] Conditions: 1 mL ethanol (17.13 mmol), 10 mL methanol, 180 °C, 400 rpm, NaOMe (mol% as indicated), [b] mol% based on ethanol substrate, [c] TON based on mmol of substrate converted to products per mmol of Re, [d] Total selectivity to products in the liquid fraction determined by GC, [e] Temp= 160 °C, [f] Temp= 200 °C

Table S2: Rhenium complexes bearing bidentate ligands for the conversion of methanol and ethanol to isobutanol

Run [a]	Complex[b]	Temperature (°C)	Ethanol Conv. (%)	TON[c] (Selectivity)[d] [Yield]		
				iBuOH	PrOH	2MeBuOH
**1**	**4**	180	-	-	-	-
**2**	**5**	180	-	-	-	-
**3**	**6**	180	-	-	-	-
**4**	**7**	180	52	124(58)[12]	70(29)[7]	26(13)[5]
**5**	**7**	200	81	283(88)[28]	40(12)[4]	0(0)[0]
**6**	**8**	180	40	88(70)[9]	40(30)[4]	0(0)[0]
**7**	**9a/9b**	180	1	-	-	-
**8**	**10**	180	26	59(45)[6]	50(36)[5]	25(19)5
**9**	**11**	180	-	-	-	-

[a] Conditions: 1 mL ethanol (17.13 mmol), 10 mL methanol, 0.1 mol% [Re], 400 rpm, 200 mol% NaOMe, 17 hrs, [b] mol% loading based on ethanol substrate, [c] TON based on mmol of substrate converted to products per mmol of Re, [d] Total selectivity to products in the liquid fraction determined by GC,

Solid analysis

The post reaction mixture (Table S1, Entry 1), was isolated by Büchner filtration and washed with toluene (100 mL). The white solid was dried *in vacuo* for 1 hour before being weighed (1.006 g). ^{1}H and ^{13}C NMR analysis was undertaken in D_2O.

Mechanistic experiments

Open system base reactions

Complex **1** (0.003 g, 0.005 mmol), was dissolved in dry, degassed methanol (2 mL) and NaOMe (0.247 g, 4.6 mmol, 1000 fold excess) was added to the solution. This was heated at reflux for 20 hours, and the product analysed by ^{31}P NMR spectroscopy.

^{31}P{^{1}H} NMR (202 MHz, MeOH): (δ, ppm) 38.66

Closed system base experiments

Complex **1** (0.007 g, 0.011 mmol) and NaOMe (0.58 g, 10.7 mmol, 1000 fold excess) were loaded in an oven dried fitted PTFE insert in a 300 mL Parr stainless steel autoclave. The autoclave was then put under a nitrogen atmosphere, sealed and heated to 180 ^{o}C for 18 hours. The autoclave was cooled using an ice-water bath, and then reattached to a Schlenk line. A sample of liquid was extracted under a backflow of nitrogen and analysed by $^{31}P\{^{1}H\}$ NMR spectroscopy. Any solid produced was analysed by ^{1}H NMR spectroscopy as detailed above.

$^{31}P\{^{1}H\}$ NMR (202 MHz, MeOH): (δ, ppm) 53.90

High temperature experiments without base

Performed as above, except no NaOMe was added into the autoclave. Analysed by ^{31}P NMR spectroscopy.

$^{31}P\{^{1}H\}$ NMR (202 MHz, MeOH): (δ, ppm) 46.26 (s), 47.98 (s), 49.84 (s)

Open system base reactions- Ethanol solvent

Complex **1** (0.028 g, 0.043 mmol), was dissolved in dry, degassed ethanol (5 mL) and NaOEt (0.29 g, 4.3 mmol, 100 fold excess) was added to the solution. This was heated at reflux for 20 hours, resulting in a golden solution. The product was analysed by $^{31}P\{^{1}H\}$ NMR spectroscopy.

$^{31}P\{^{1}H\}$ NMR (202 MHz, EtOH): (δ, ppm) 51.18 (br, s), 39.01 (s)

Open system base tests- Catalysts 4 + 5

Performed as above, with 0.0038 mmol complex and 0.381 mmol (100 fold excess) NaOMe. Samples were heated at reflux for 3 days. Analysis by ^{31}P NMR spectroscopy.

Complex **6**: $^{31}P\{^{1}H\}$ NMR (202 MHz, MeOH): (δ, ppm) -33.65 (t, $^{2}J_{pp}$= 17.1 Hz), -46.11 (t, $^{2}J_{pp}$= 17.0 Hz)

Complex **7**: $^{31}P\{^{1}H\}$ NMR (202 MHz, MeOH): (δ, ppm) -33.64 (t, $^{2}J_{pp}$= 17.0 Hz), -46.10 (t, $^{2}J_{pp}$= 17.0 Hz)

Spectra

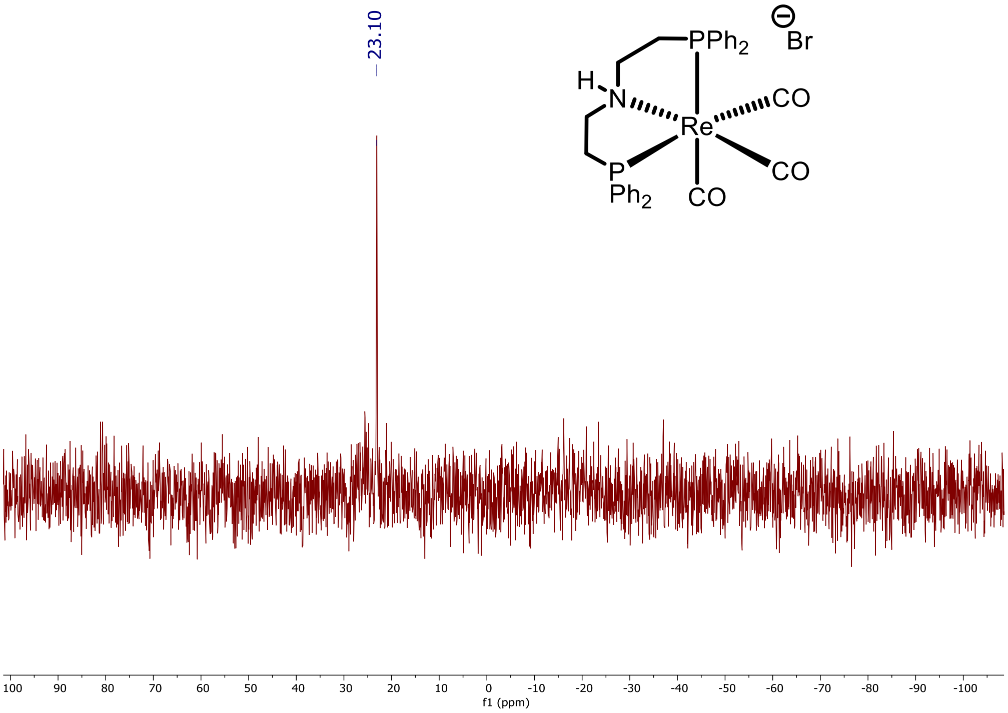

Figure S1: $^{31}P\{^{1}H\}$ NMR spectrum of complex **2** in $CDCl_3$ (122 MHz).

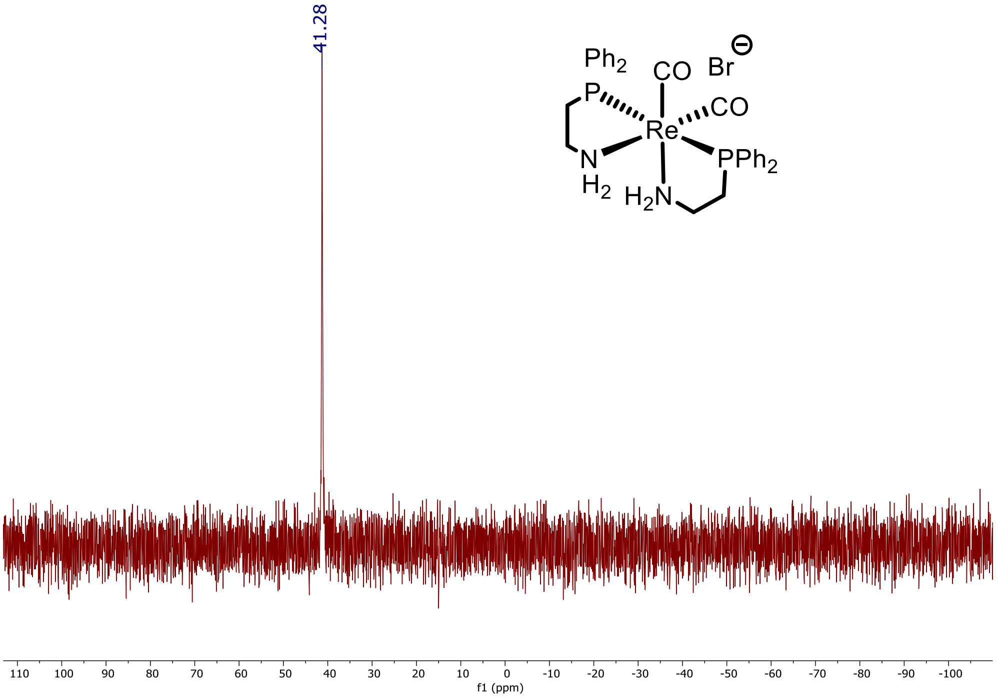

Figure S2: $^{31}P\{^{1}H\}$ NMR spectrum of complex **7** in $CDCl_3$ (122 MHz).

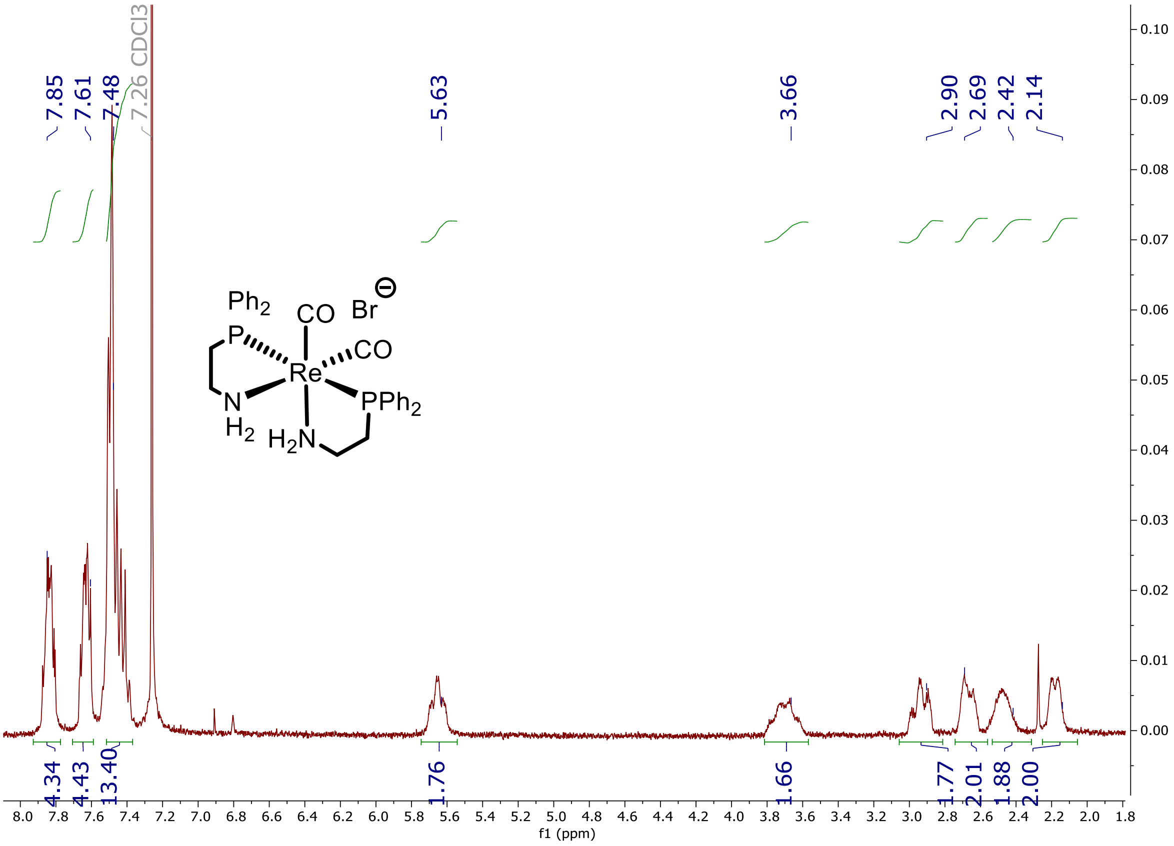

Figure S3: ^{1}H NMR spectrum of complex **7** in $CDCl_3$ (300 MHz).

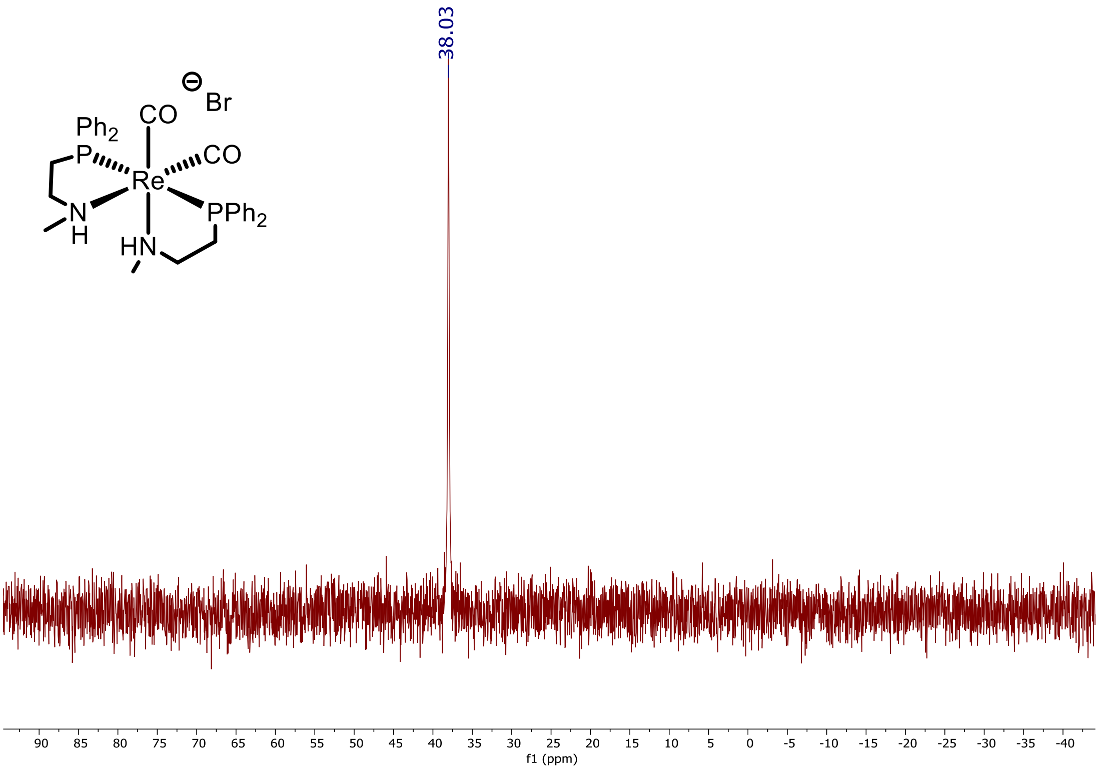

Figure S4: $^{31}P\{^{1}H\}$ NMR spectrum of complex **8** in $CDCl_3$ (122 MHz).

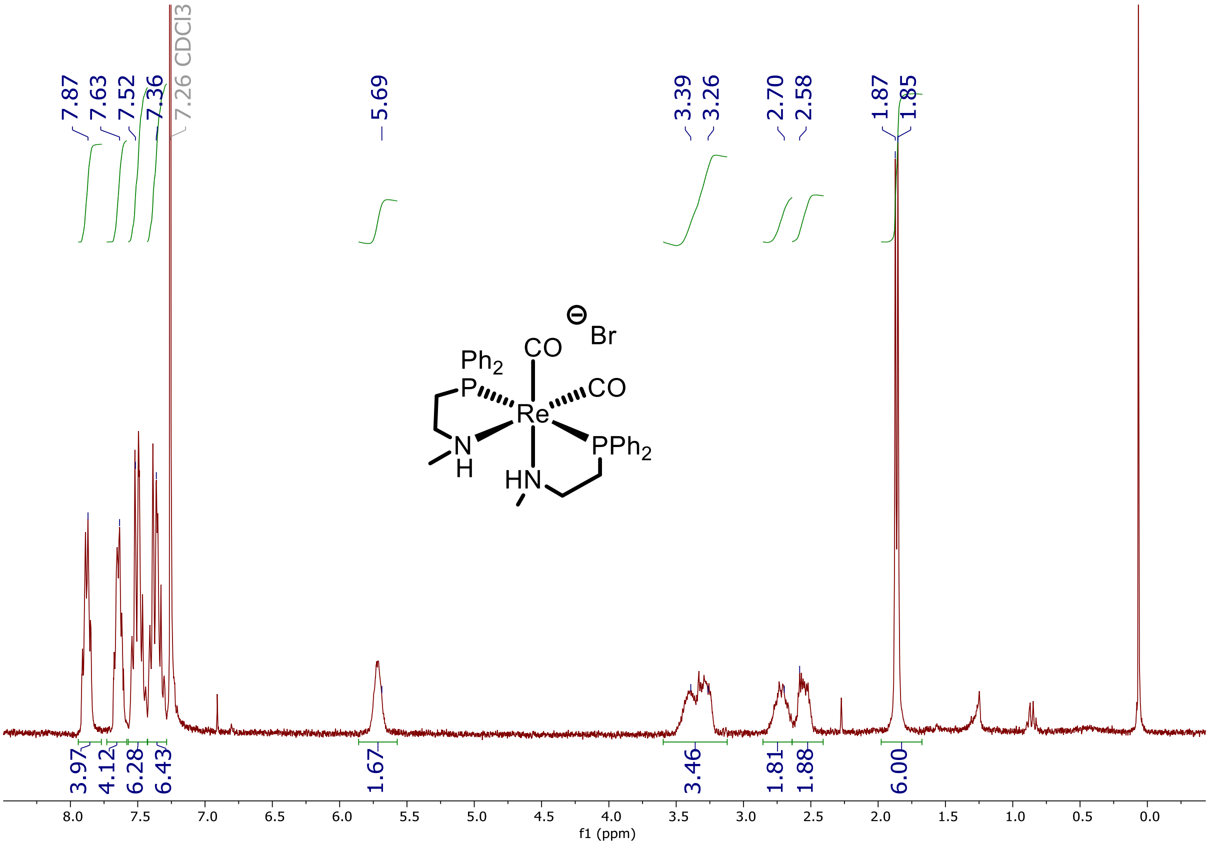

Figure S5: ^{1}H NMR spectrum of complex **8** in $CDCl_3$ (300 MHz).

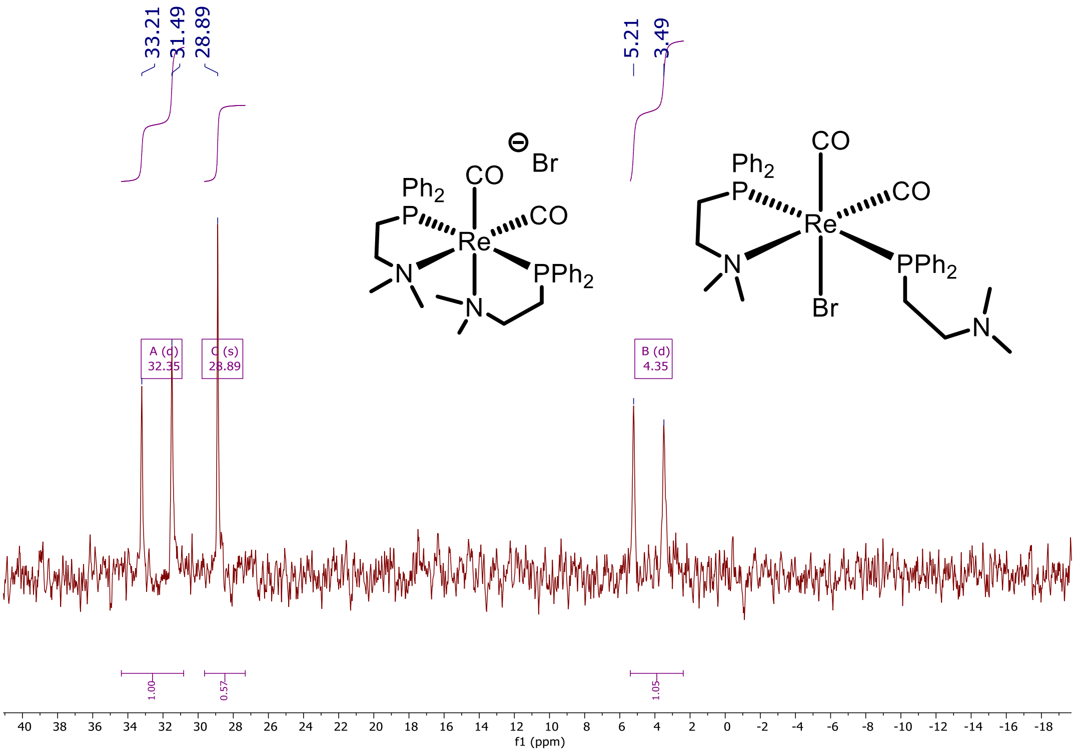

Figure S6: $^{31}P\{^1H\}$ NMR spectrum of complex **9a/9b** in $CDCl_3$ (122 MHz).

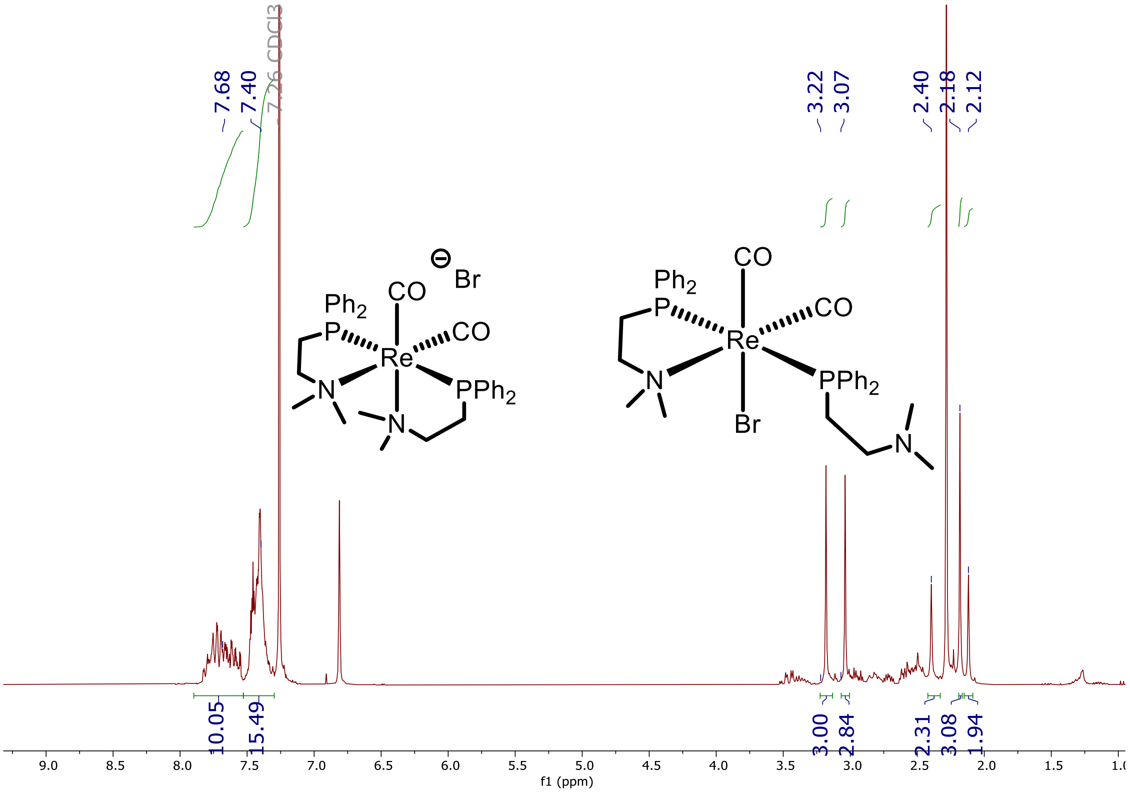

Figure S7: ^{1}H NMR spectrum of complex **9a/9b** in $CDCl_3$ (300 MHz).

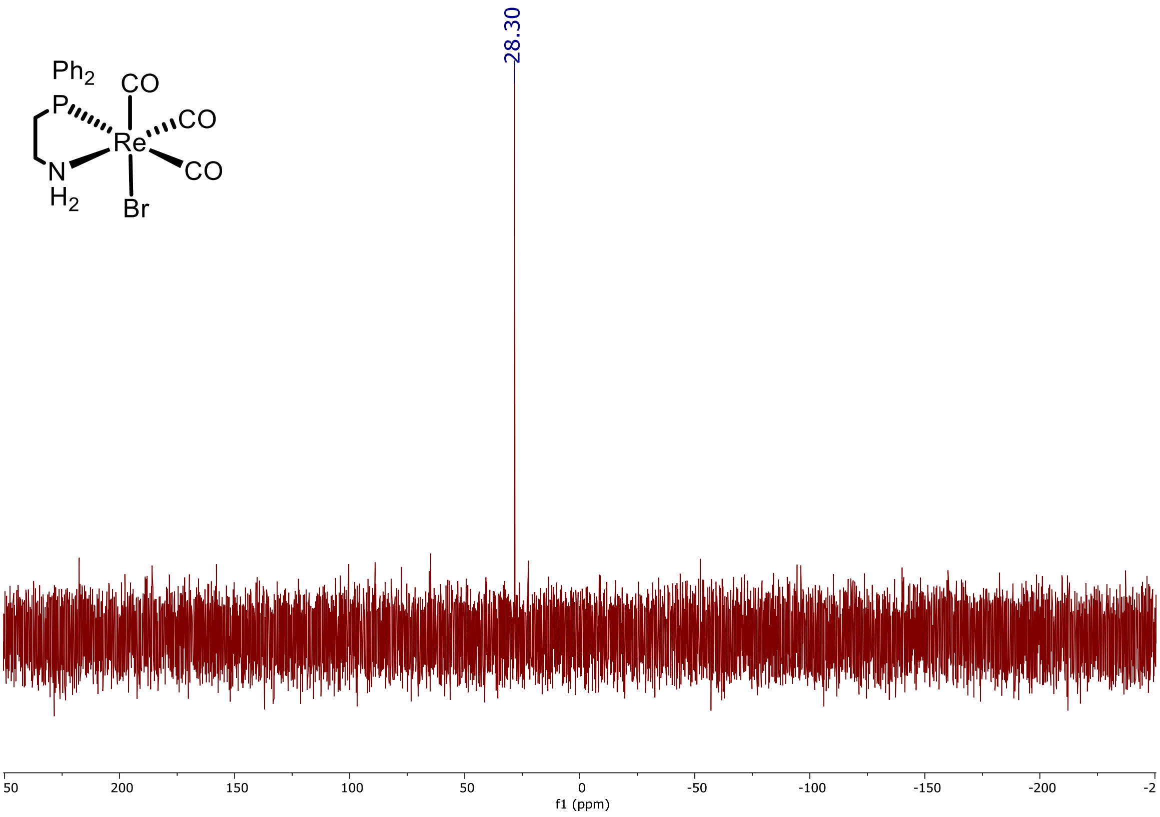

Figure S8: $^{31}P\{^{1}H\}$ NMR spectrum of complex **10** in $CDCl_3$ (122 MHz).

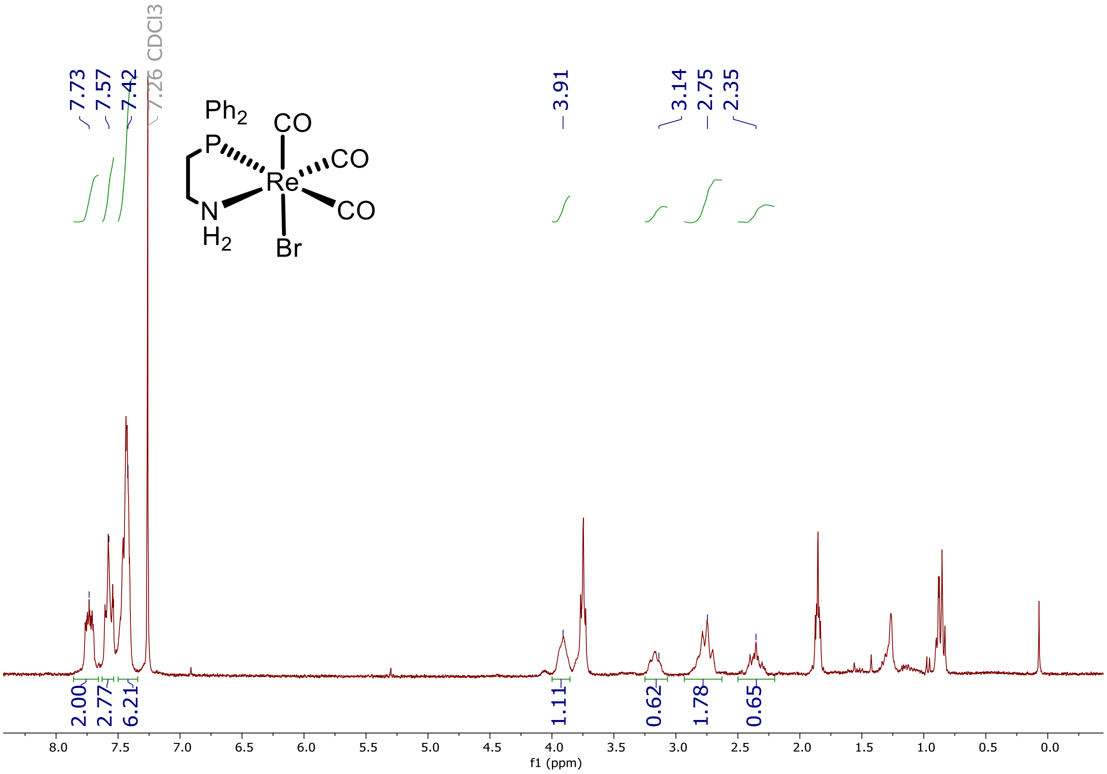

Figure S9: ^{1}H NMR spectrum of complex **10** in $CDCl_3$ (300 MHz).

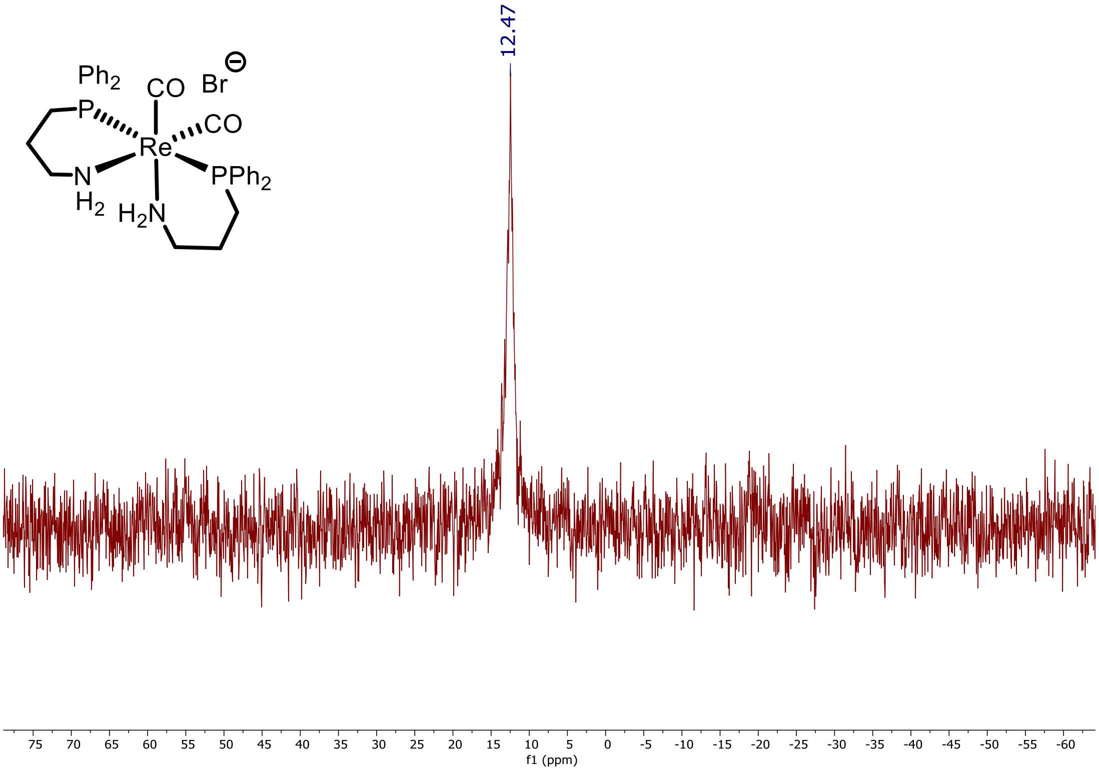

Figure S10: $^{31}P\{^{1}H\}$ NMR spectrum of complex **11** in $CDCl_3$ (122 MHz).

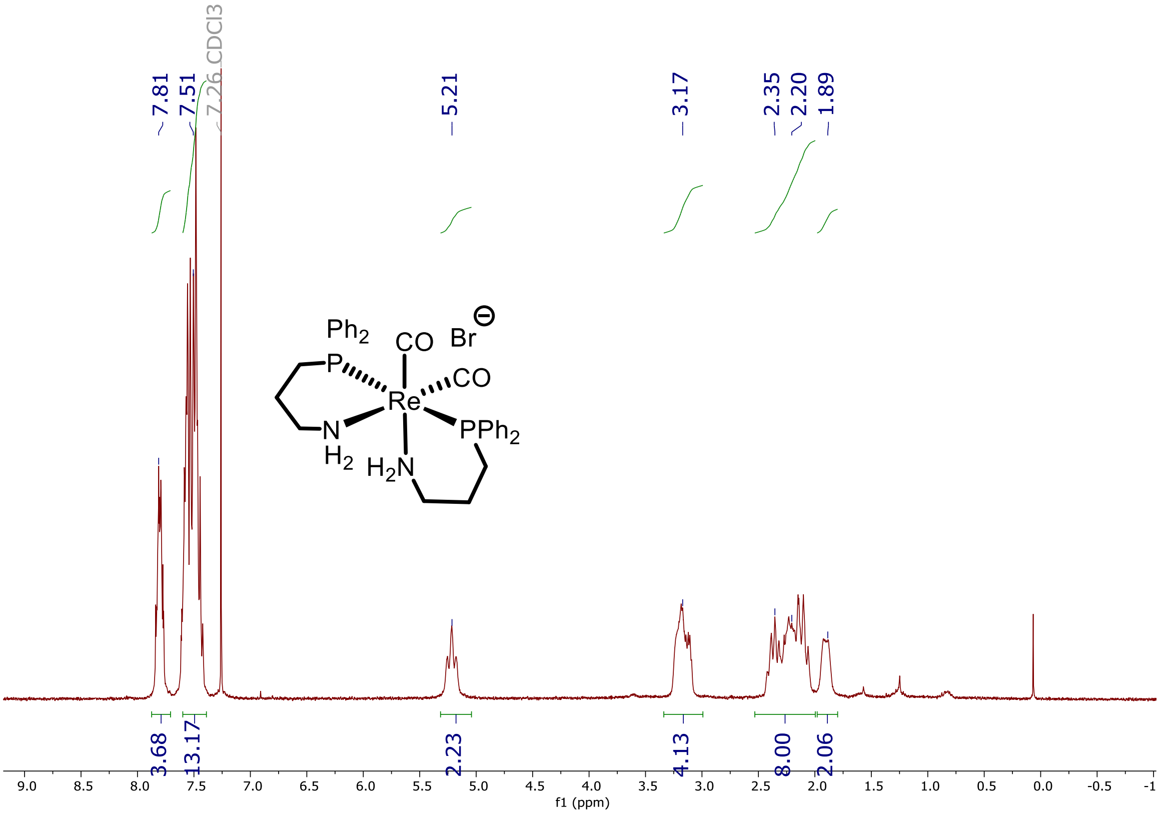

Figure S11: ^{1}H NMR spectrum of complex **11** in $CDCl_3$ (300 MHz).

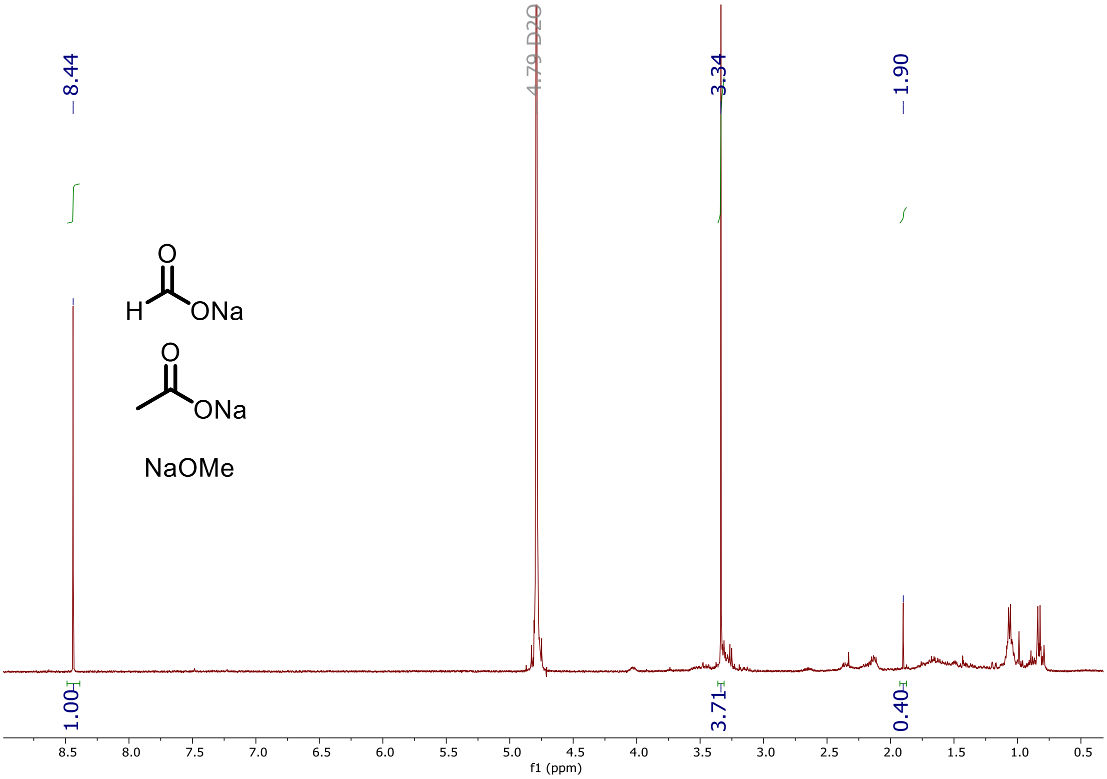

Figure S12: ^{1}H NMR spectrum of the solid produced by Run 1 (Table S1) in D_2O (301 MHz) - sodium formate seen at 8.44 ppm, sodium methoxide at 3.34 and sodium acetate at 1.90 ppm.

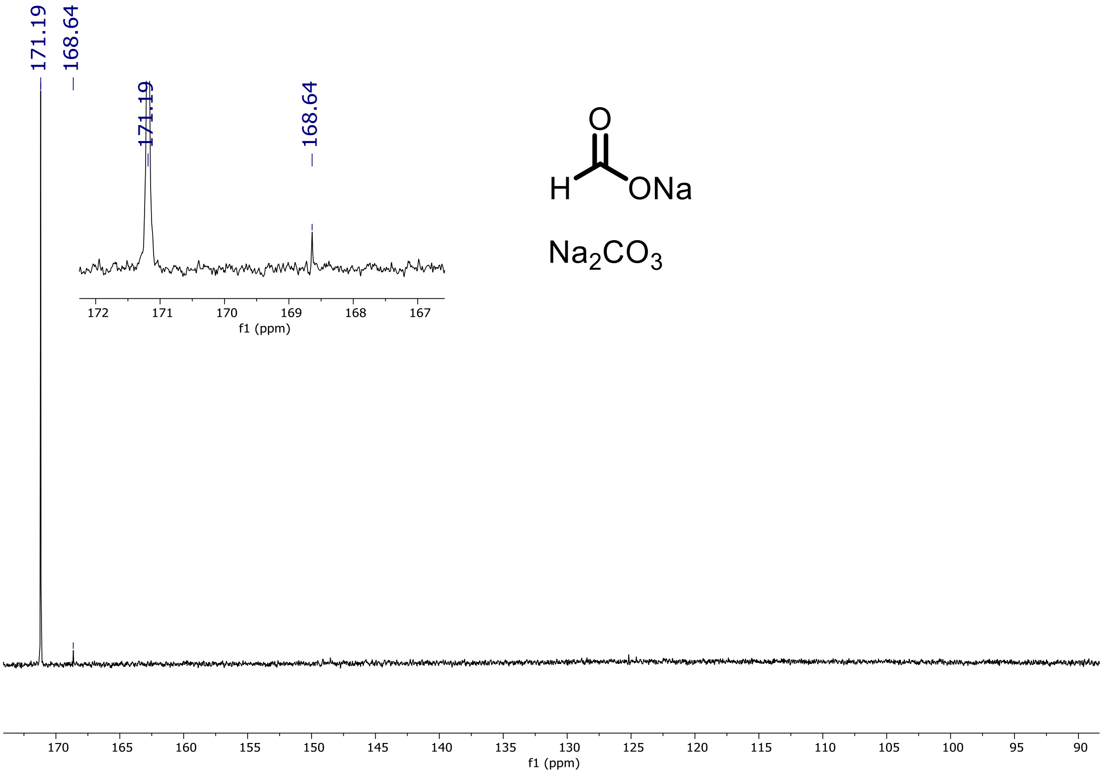

Figure S13: ^{13}C NMR spectrum of solid produced by Run 3 (Table S1) in D_2O (126 MHz). Inset: expanded section of the formate/ carbonate region. Sodium formate seen at 171 ppm and sodium carbonate at 169 ppm.

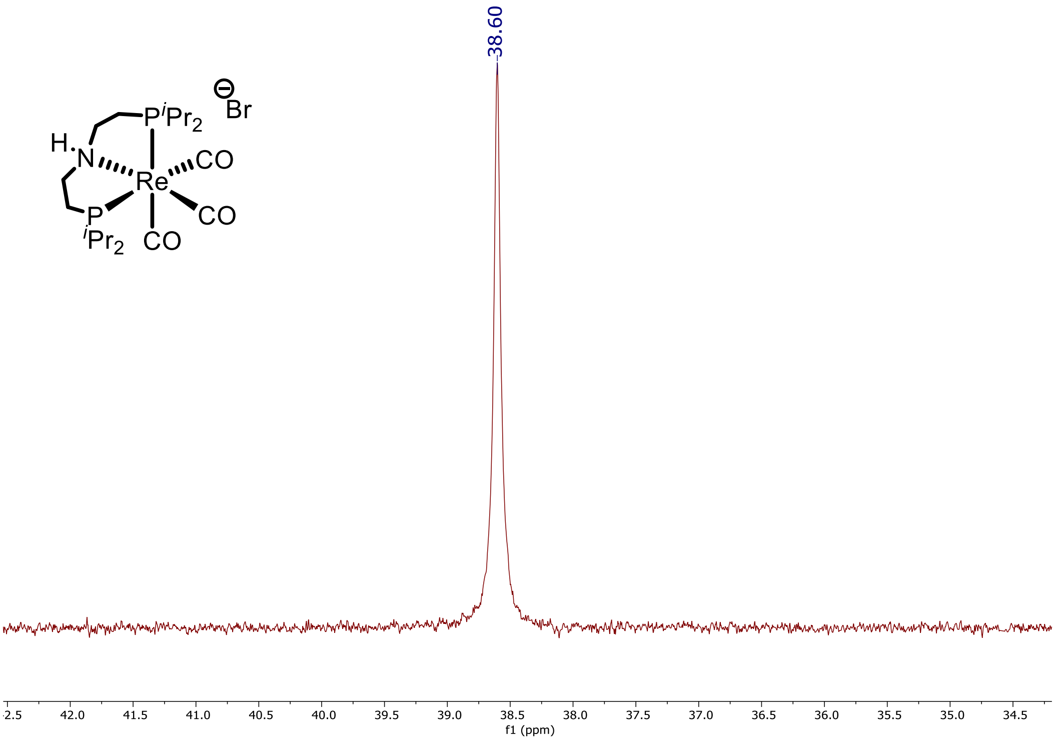

Figure S14: $^{31}P\{^1H\}$ NMR spectrum (unlocked, MeOH, 188 MHz) of complex **1** after having been dissolved in MeOH for 4 days.

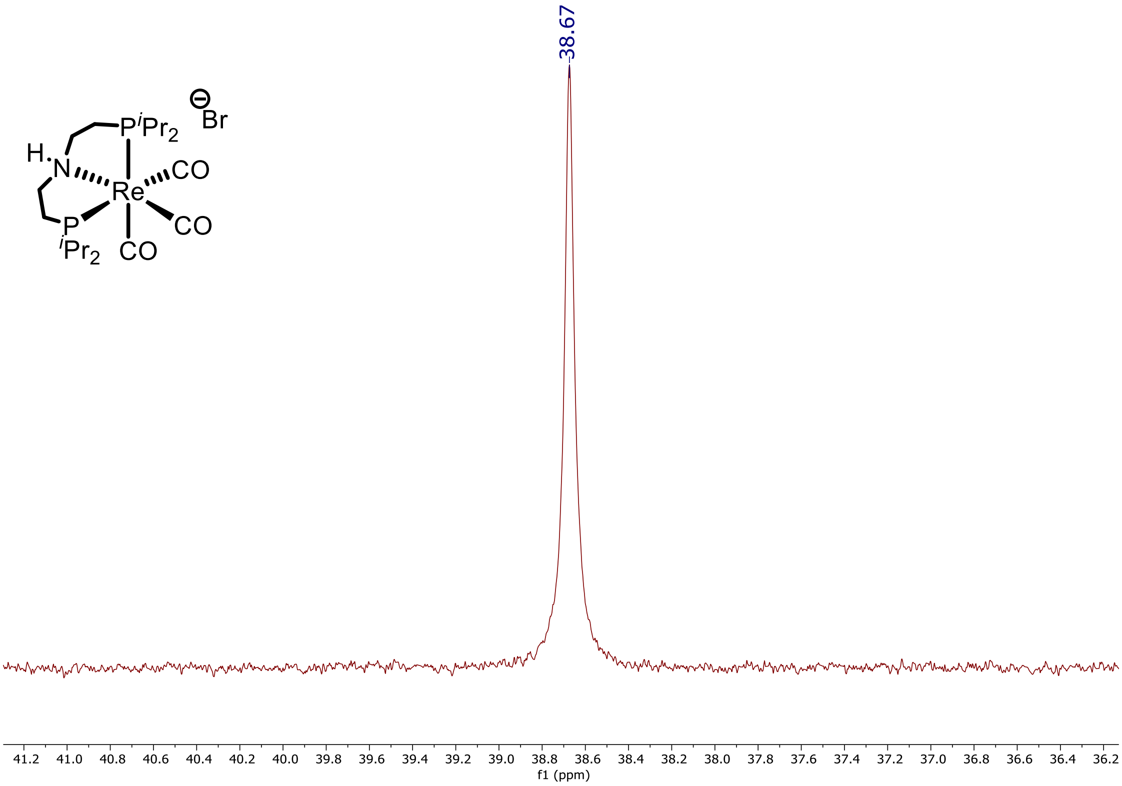

Figure S15: $^{31}P\{^1H\}$ NMR spectrum (unlocked, 188 MHz, MeOH) of complex **1** after the addition of 100 eqv. NaOMe to the above sample.

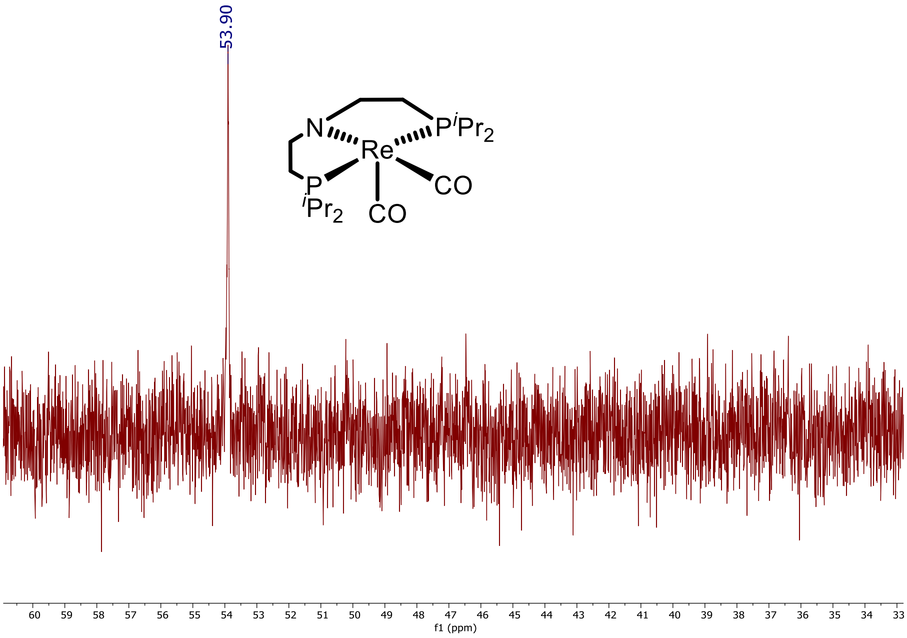

Figure S16: $^{31}P\{^1H\}$ NMR spectrum (unlocked, 188 MHz, MeOH) of complex **1** after heating to 180 °C in a sealed autoclave with 1000 eqv. NaOMe.

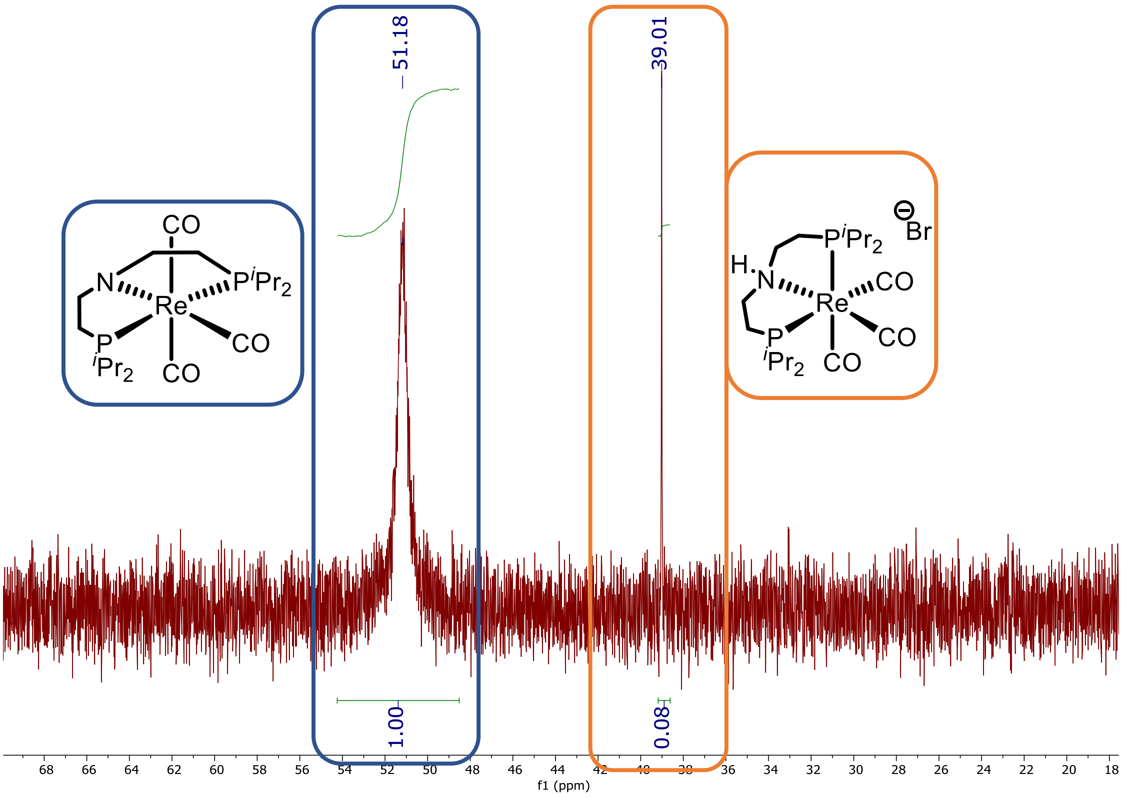

Figure S17: $^{31}P\{^{1}H\}$ NMR spectrum (unlocked, 188 MHz, EtOH) of complex **1** after heating to reflux in an ethanol solution.

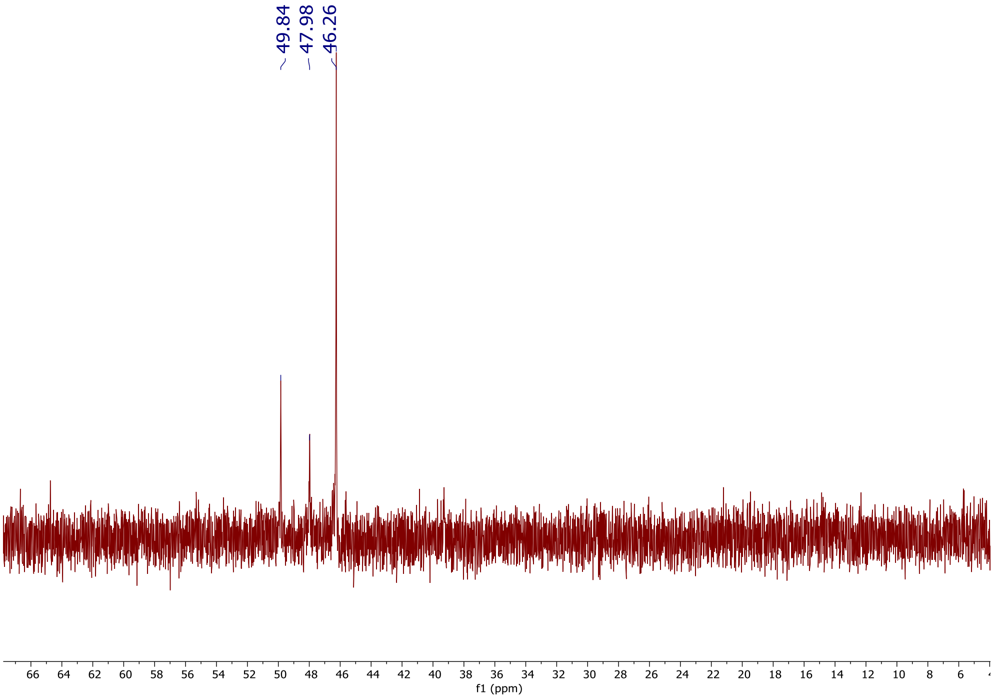

Figure S18: $^{31}P\{^{1}H\}$ NMR spectrum (unlocked, 188 MHz, MeOH) of complex **1** after heating to 180 °C in a sealed autoclave in the absence of base.

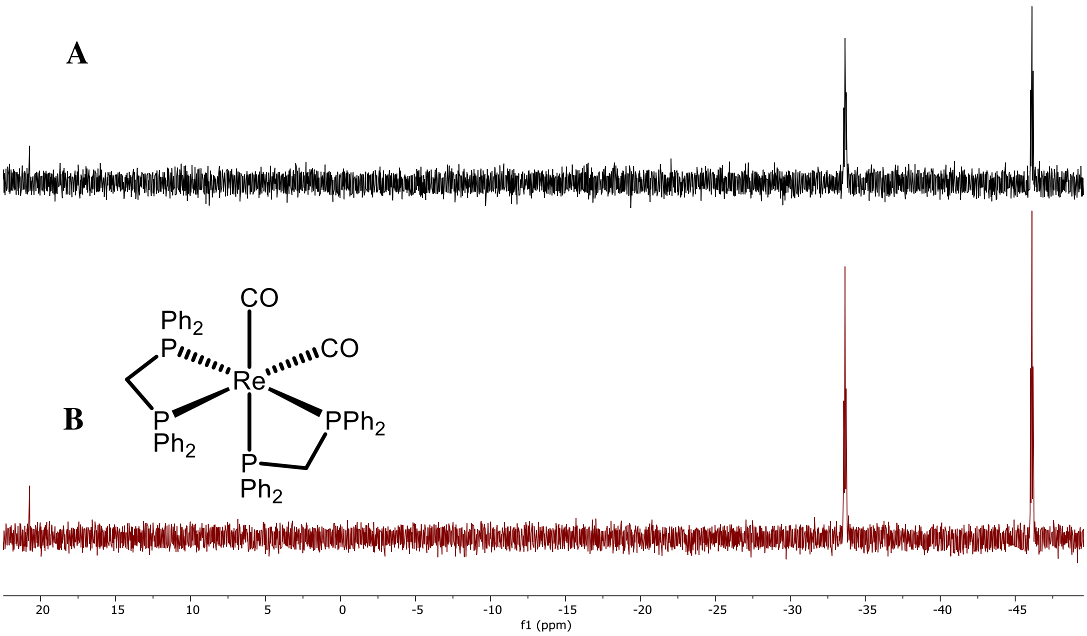

Figure S19: $^{31}P\{^{1}H\}$ NMR spectra (unlocked, 188 MHz, MeOH) of complex **4 (A)** and **5 (B)** after being heated to reflux in methanol with 100 eqv. NaOMe.

Crystallographic data

X-ray diffraction experiments on **7, 10** and **11** were carried out at 100(2) K on a Bruker APEX II diffractometer using Mo-Kα radiation (λ = 0.71073 Å). Data collections were performed using a CCD area detector. Intensities were integrated in SAINT[9] and absorption corrections based on equivalent reflections were applied using SADABS.[10] Structures **7**, **10** and **11** were solved using ShelXT[11] and refined by full matrix least squares against *F*2 in ShelXL[12,13] using Olex2.[14] All of the non-hydrogen atoms were refined anisotropically. While all of the hydrogen atoms were located geometrically and refined using a riding model, apart from the none-disordered N-H protons in **7**, **10** and **11** which were located in the difference map and refined with isotropic displacement parameters Uiso(H) = 1.2Ueq(N). In the case of **11** there are two molecules present in the asymmetric unit and one displayed disorder, disorder was also present in one of the bromide counter ions and one of the water solvent molecules. The occupancies of the fragments were determined by refining them against a free variable with the sum of the two sites set to equal 1. SADI and SIMU were used to maintain sensible geometries and thermal parameters. Crystal structure and refinement data are given in Table S3. Crystallographic data for compounds **7**, **10** and **11** have been deposited with the Cambridge Crystallographic Data Centre as supplementary publication CCDC 2072199-2072201. Copies of the data can be obtained free of charge on application to CCDC, 12 Union Road, Cambridge CB2 1EZ, UK [fax(+44) 1223 336033, e-mail: deposit@ccdc.cam.ac.uk].

Table S3: Crystal data and structure refinement for **7**, **10** and **11**

**Complex**	**7**	**10**	**11**
**CCDC number**	2072199	2072200	2072201
**Empirical formula**	$C_{34}H_{42}BrN_2O_3P_2Re$	$C_{18}H_{18}BrCl_2NO_3PRe$	$C_{32}H_{38}BrN_2O_3P_2Re$
**Formula weight**	854.74	664.31	826.69
**Temperature/K**	99.98	99.99	100.11
**Crystal system**	monoclinic	monoclinic	triclinic
**Space group**	$P2_1/c$	$P2_1/c$	P-1
**a/Å**	9.7634(2)	11.3995(4)	11.4430(2)
**b/Å**	21.8195(4)	11.7397(4)	17.3552(4)
**c/Å**	15.8673(3)	15.4962(6)	18.1242(4)

**α/°**	90	90	66.2390(10)
**β/°**	97.3290(10)	92.6836(19)	73.4960(10)
**γ/°**	90	90	74.8580(10)
**Volume/Å^3**	3352.63(11)	2071.53(13)	3114.08(12)
**Z**	4	4	4
**ρ_{calc}g/cm^3**	1.693	2.130	1.763
**μ/mm^{-1}**	4.948	8.149	5.324
**F(000)**	1696.0	1264.0	1632.0
**Crystal size/mm^3**	0.538 × 0.16 × 0.04	0.339 × 0.248 × 0.176	0.43 × 0.2 × 0.08
**Radiation**	MoKα (λ = 0.71073)	MoKα (λ = 0.71073)	MoKα (λ = 0.71073)
**2Θ range for data collection/°**	3.734 to 60.338	4.354 to 56.558	2.6 to 60.144
**Index ranges**	-13 ≤ h ≤ 13, -30 ≤ k ≤ 30, -22 ≤ l ≤ 22	-15 ≤ h ≤ 15, -15 ≤ k ≤ 15, -20 ≤ l ≤ 20	-16 ≤ h ≤ 13, -24 ≤ k ≤ 24, -25 ≤ l ≤ 25
**Reflections collected**	56044	30202	68081
**Independent reflections**	9928 [R_{int} = 0.0341, R_{sigma} = 0.0243]	5145 [R_{int} = 0.0335, R_{sigma} = 0.0226]	18222 [R_{int} = 0.0562, R_{sigma} = 0.0574]
**Data/restraints/parameters**	9928/0/406	5145/0/252	18222/94/822
**Goodness-of-fit on F^2**	1.058	1.045	1.003
**Final R indexes [I>=2σ (I)]**	R_1 = 0.0217, wR_2 = 0.0439	R_1 = 0.0170, wR_2 = 0.0390	R_1 = 0.0330, wR_2 = 0.0658
**Final R indexes [all data]**	R_1 = 0.0271, wR_2 = 0.0453	R_1 = 0.0190, wR_2 = 0.0396	R_1 = 0.0628, wR_2 = 0.0754
**Largest diff. peak/hole / e Å^{-3}**	0.90/-1.33	1.67/-0.67	1.53/-1.85

References

1 A. Habtemariam, B. Watchman, B. S. Potter, R. Palmer, S. Parsons, A. Parkin and P. J. Sadler, *J. Chem. Soc. Dalt. Trans.*, 2001, 1306–1318.

2 P. Piehl, M. Peña-López, A. Frey, H. Neumann and M. Beller, *Chem. Commun.*, 2017, **53**, 3265–3268.

3 D. Wei, O. Sadek, V. Dorcet, T. Roisnel, C. Darcel, E. Gras, E. Clot and J.-B. Sortais, *J. Catal.*, 2018, **366**, 300–309.

4 N. Lazarova, S. James, J. Babich and J. Zubieta, *Inorg. Chem. Commun.*, 2004, **7**, 1023–1026.

5 D. Mori, H. Kimura, H. Kawashima, Y. Yagi, K. Arimitsu, M. Ono and H. Saji, *Bioorganic Med. Chem.*, 2019, **27**, 4200–4210.

6 S. W. Carr, B. L. Shaw and M. Thornton-Pett, *J. Chem. Soc. Dalt. Trans.*, 1987, 1763–1768.

7 A. M. Bond, R. Colton, D. G. Humphrey, P. J. Mahon, G. A. Snook, V. Tedesco and J. N. Walter, *Organometallics*, 1998, **17**, 2977–2985.

8 G. A. Carriedo, M. L. Rodríguez, S. García-Granda and A. Aguirre, *Inorganica Chim. Acta*, 1990, **178**, 101–106.

9 *Bruker, SAINT+ v8.38A Integration Engine, Data Reduction Software, Bruker Analytical X-ray Instruments Inc., Madison, WI, USA, 2015.*, .

10 *Bruker, SADABS 2014/5, Bruker AXS area detector scaling and absorption correction, Bruker Analytical X-ray Instruments Inc., Madison, Wisconsin, USA, 2014/5.*, .

11 *G. M. Sheldrick, Acta Crystallographica a-Foundation and Advances, 2015, 71, 3-8*, .

12 *G. M. Sheldrick, Acta Crystallogr. C, 2015, 71, 3-8.*, .

13 *G. M. Sheldrick, Acta Crystallogr., Sect. A: Found. Crystallogr., 2008, 64, 112-122.*, .

14 *O. V. Dolomanov, L. J. Bourhis, R. J. Gildea, J. A. K. Howard and H. Puschmann, J. Appl. Crystallogr., 2009, 42, 339-341.*, .
